# Effects of Education and Income on Incident Type 2 Diabetes and Cardiovascular Diseases: a Dutch Prospective Study

**DOI:** 10.1007/s11606-022-07548-8

**Published:** 2022-04-13

**Authors:** Ming-Jie F. Duan, Yinjie Zhu, Louise H. Dekker, Jochen O. Mierau, Eva Corpeleijn, Stephan J.L. Bakker, Gerjan Navis

**Affiliations:** 1 Department of Internal Medicine, Division of Nephrology, University Medical Center Groningen, University of Groningen, Groningen, The Netherlands; 2 National Institute for Public Health and the Environment, Bilthoven, The Netherlands; 3 Aletta Jacobs School of Public Health, University of Groningen, Groningen, The Netherlands; 4 Faculty of Economics and Business, University of Groningen, Groningen, The Netherlands; 5 Department of Epidemiology, University Medical Center Groningen, University of Groningen, Groningen, The Netherlands

**Keywords:** socioeconomic status, education, income, type 2 diabetes, cardiovascular diseases, health disparities, status inconsistency

## Abstract

**Background:**

Education and income, as two primary socioeconomic indicators, are often used interchangeably in health research. However, there is a lack of clear distinction between these two indicators concerning their associations with health.

**Objective:**

This study aimed to investigate the separate and combined effects of education and income in relation to incident type 2 diabetes and cardiovascular diseases in the general population.

**Design and Participants:**

Participants aged between 30 and 65 years from the prospective Dutch Lifelines cohort study were included. Two sub-cohorts were subsequently created, including 83,759 and 91,083 participants for a type 2 diabetes cohort and a cardiovascular diseases cohort, respectively.

**Main Measures:**

Education and income level were assessed by self-report questionnaires. The outcomes were incident type 2 diabetes and cardiovascular diseases (defined as the earliest non-fatal cardiovascular event).

**Key Results:**

A total of 1228 new cases of type 2 diabetes (incidence 1.5%) and 3286 (incidence 3.6%) new cases of cardiovascular diseases were identified, after a median follow-up of 43 and 44 months, respectively. Low education and low income (<1000 euro/month) were both positively associated with a higher risk of incident type 2 diabetes (OR 1.24 [95%CI 1.04–1.48] and OR 1.71 [95%CI 1.30–2.26], respectively); and with a higher risk of incident cardiovascular diseases (OR 1.15 [95%CI 1.04–1.28] and OR 1.24 [95%CI 1.02–1.52], respectively); independent of age, sex, lifestyle factors, BMI, clinical biomarkers, comorbid conditions at baseline, and each other. Results from the combined associations of education and income showed that within each education group, a higher income was associated with better health; and similarly, a higher education was associated with better health within each income group, except for the low-income group.

**Conclusions:**

Education and income were both independently associated with incident type 2 diabetes and cardiovascular diseases. The combined associations of these two socioeconomic indicators revealed that within each education or income level, substantial health disparities existed across strata of the other socioeconomic indicator. Education and income are two equally important socioeconomic indicators in health, and should be considered simultaneously in health research and policymaking.

**Supplementary Information:**

The online version contains supplementary material available at 10.1007/s11606-022-07548-8.

## INTRODUCTION

Health disparities related to non-communicable diseases persist across socioeconomic strata. Abundant evidence has demonstrated that people with low socioeconomic status (SES) are disproportionately affected by higher risks of all-cause mortality, the metabolic syndrome, type 2 diabetes, and cardiovascular diseases.^1–4^ It has been suggested that limited access to health and health-care resources, chronic stress, unhealthy lifestyle, and exposure to pollutants were found to play an important role in explaining the adverse health outcomes associated with low SES.^5^

Education and income are two primary components of SES. However, a clear distinction between these two socioeconomic indicators is often lacking.^3, 6, 7^ Many studies on health disparities only considered one of t0hem,^7^ while some other studies focused on an aggregate measure of SES derived from multivariate statistics.^8, 9^ Research often made references to one indicator to corroborate findings into the other. There is increasing awareness that education and income should not be used interchangeably, since they capture different dimensions of health-related resources and may impact on health through different pathways.^6, 7^ As the main upstream determinants of health outcomes, research is needed to clarify the differences between education and income concerning their associations with health outcomes.

In public health practice, the inconsistent use of education and income may result in inaccurate identification of socioeconomically vulnerable groups, since people do not always hold a matching socioeconomic position.^8^ It has been suggested that having such status inconsistency carries its own health risks. However, to date, only a few studies have explored such health disparities within different socioeconomic strata.^10–13^ It is therefore also important to assess how different combinations of education and income levels are associated with health outcomes.

Therefore, using a large Dutch population cohort, this study aimed to evaluate the effects of education and income — separately and jointly using a combined indicator — on incident type 2 diabetes and cardiovascular diseases. Specifically, this study aimed to address how education and income may contribute to the short-term inequities in these two health outcomes.

## METHODS

### Cohort Design

The Lifelines cohort is a multidisciplinary prospective population-based cohort study that uses a unique three-generation design to study the health and health-related behaviors of 167,729 persons living in the north of The Netherlands. It employs a broad range of investigative procedures in assessing the biomedical, socio-demographic, behavioral, and physical factors, which contribute to health and disease of the general population. Before study entry, a signed informed consent form was obtained from each participant. The Lifelines study is conducted according to the principles of the Declaration of Helsinki and approved by the Medical Ethics Committee of the University Medical Center Groningen, The Netherlands. The overall design and rationale of the study have been described in detail elsewhere.^14, 15^

After the baseline assessment (T1, years 2007 to 2013), all participants were invited for new rounds of assessments approximately every 5 years. In between assessments, follow-up questionnaires were completed approximately once every 1.5–2.5 years (Supplementary Figure S1). The current analysis used data from the baseline assessment T1 and the second assessment T4, as well as the two follow-ups (T2 and T3) in between. Currently, the third round of assessment is on-going. Comprehensive physical examinations, biobanking of blood and urine, and questionnaires were conducted at T1 and T4. Follow-up questionnaires for status of type 2 diabetes and cardiovascular diseases were issued to participants at T2, T3, and T4.

### Study Population

For this study, we included all participants aged between 30 and 65 years. We subsequently created two sub-cohorts from those included participants, with one for type 2 diabetes and the other for cardiovascular diseases. For the diabetes cohort, we included participants who were free of diabetes at baseline, and further excluded participants who had no follow-up data to determine status of diabetes. We also excluded participants who reported the development of type 1 diabetes or gestational diabetes during the follow-ups. For the cardiovascular diseases cohort, we included participants who were free of cardiovascular diseases at baseline, and further excluded participants who had no follow-up data to determine status of cardiovascular diseases. Participants who had less than 1 year of follow-up after baseline were also excluded. In order to avoid massive imputation, we additionally excluded participants who had no available data on education level and BMI at baseline for both sub-cohorts. This led to an additional exclusion of approximately 0.5% of the study population, which was not expected to influence our results. In total, 83,759 and 91,083 participants were included and analyzed in the diabetes cohort and the cardiovascular diseases cohort, respectively. Supplementary Figure S2 shows the study flow chart.

### Data Collection

#### Ascertainment of Incident Type 2 Diabetes and Cardiovascular Diseases

Incident type 2 diabetes and cardiovascular diseases were assessed by self-report questionnaires at the two follow-ups (T2 and T3) and the second assessment (T4). Additionally, we assessed incident cases based on blood measurements and pathology on electrocardiograms, which were available at the second assessment (T4). For type 2 diabetes, an incident case was considered as fasting blood glucose ≥ 7.0 mmol/L or HbA_1c_ ≥ 6.5%.^16^ For cardiovascular diseases, the primary outcome was defined as the earliest non-fatal major cardiovascular event, including stroke (ischemic and hemorrhagic), myocardial infarction, heart failure, percutaneous transluminal coronary angioplasty surgery, and coronary artery bypass grafting surgery.^17^ Secondary outcome was a composite of death from any cause and non-fatal major cardiovascular event as described above. However, data on prescribed medication was not available during follow-ups. Data of medical records, causes of death, and the precise time of diagnosis were also not available.

#### Assessment of Education and Income Levels

Education and income levels were assessed by self-report questionnaires (Supplementary Table S1). Highest education level was categorized according to the International Standard Classification of Education (ISCED): (1) low (level 0, 1, or 2); (2) middle (level 3 or 4); and (3) high (level 5 or 6).^18^ Income level was based on monthly household net income and was categorized as (1) low (<1000 euro/month); (2) lower-middle (1000–2000 euro/month); (3) upper-middle (2000–3000 euro/month); (3) high (>3000 euro/month); and (4) do not know/prefer not to answer.

#### Clinical Measurements

Blood samples were collected by venipuncture in a fasting state and serum levels of glucose, HbA_1c_, HDL-cholesterol, total cholesterol, and triglycerides were analyzed. Measurements of blood pressure, 12-lead electrocardiograms, and anthropometry were made by trained research staff following standardized protocols. These measurements were performed without shoes and heavy clothing. Body mass index (BMI) was calculated as weight in kilograms divided by the square of height in meters. Hypertension status was defined as (1) hypertensive medication use (ATC codes C02, C03, C07, C08, C09); (2) systolic blood pressure ≥ 140 mmHg; or (3) diastolic blood pressure ≥ 90 mmHg.^19^

#### Assessment of Other Baseline Covariates

Age, smoking status (never, former, and current), and TV watching time were assessed by self-administered questionnaires. Physical activity level was assessed by the validated Short QUestionnaire to ASsess Health-enhancing physical activity (SQUASH), from which non-occupational moderate-to-vigorous physical activities (MVPA) were calculated in minutes per week.^20^ Dietary intake was assessed by a validated 110-item semi-quantitative self-administered food frequency questionnaire (FFQ).^21^ Macro- and micro-nutrients intake was calculated from the FFQ data according to the 2011 Dutch Food Composition Table (NEVO).^22^ The Lifelines Diet Score (LLDS) was calculated to evaluate the relative diet quality of each participant. The development of the LLDS has been described in detail elsewhere.^23^

### Statistical Analysis

Associations of income and education with incident type 2 diabetes and cardiovascular diseases were estimated by logistic regression models, and results were shown as odds ratios (ORs) with 95% confidence intervals. For evaluation of the separate effects of education and income, these two socioeconomic indicators were singly and mutually adjusted in the models. An interaction term between education and income was also fit into the model to test possible effect modification. For evaluation of the combined effects of education and income, these two socioeconomic indicators were combined into twelve groups, e.g., a group of participants had high education and lower-middle income. The associations of these combined groups of education and income with incident type 2 diabetes and cardiovascular diseases were subsequently estimated. For all estimations, models were adjusted in a two-step manner: (1) basic model: age and sex; (2) multivariate model: age and sex from basic model, plus lifestyle behaviors (smoking status, TV watching time, non-occupational MVPA, total energy intake, LLDS, and alcohol intake), BMI, and clinical biomarkers (HDL-cholesterol, triglycerides, and blood pressure). For cardiovascular diseases, in the multivariate model, we additionally adjusted total cholesterol level and comorbid conditions at baseline (atrial fibrillation and diabetes). In all models, age was adjusted as a categorical variable, i.e., 30–39, 40–49, 50–59, and 60–65 years. Before estimation, values of HDL-cholesterol, triglycerides, BMI, total energy intake, total cholesterol, and blood pressure were log-transformed to improve normality. We also assessed the associations with adjustments for different domains of modifiable risk factors separately. Additionally, we determined the contribution of each modifiable risk factor in explaining the associations of income and education with incident type 2 diabetes and cardiovascular diseases by calculating the percentage of attenuation in the ORs after additional adjustment for another modifiable risk factor, in comparison to the previous reference model, namely 100%×(OR_ref_−OR_new_)/OR_ref_.

Multiple imputation by chained equations was performed (creating 25 imputed datasets) to deal with missing data for income level (including both missing value and participants who responded “do not know” or “prefer not to answer”), LLDS, total energy intake, alcohol intake, non-occupational MVPA, and smoking status.^24^ These variables all had missing data more than 1%. All statistical analyses were conducted using STATA (version 13.1; StataCorp, College Station, TX).

### Sensitivity Analysis

First, we repeated our analysis without imputation of income level for those who responded “do not know” or “prefer not to answer.” Instead, we recoded them as a single category in the income variable. Second, we evaluated the potential effect modifications by sex, age, unemployment status, comorbid conditions at baseline (cancer and cardiovascular diseases), and diabetes status at baseline and during follow-ups (for cardiovascular diseases), by additionally including an interaction term with education or income in the model. Third, for cardiovascular diseases, we performed a separate analysis, in which we adjusted the SCORE2 risk prediction algorithms according to the European Society of Cardiology.^25^ For type 2 diabetes, we additionally analyzed a composite outcome of incident type 2 diabetes and death from any cause, to gain insights into how death events during follow-ups may influence the results.

## RESULTS

Baseline characteristics are shown in Table 1 and Supplementary Table S2. Approximately 28%, 40%, and 32% of participants reported having low, middle, and high education, respectively. For household net income, approximately 3%, 18%, 30%, and 33% of participants reported having low (<1000 euro), lower-middle (1000–2000 euro), upper-middle (2000–3000 euro), and high (>3000 euro) level of income per month, respectively; approximately 15% of participants did not disclose their income level. These numbers were comparable with the national-level data in The Netherlands, e.g., approximately 10% low-income households, and approximately 28% and 30% of the population had high and low education, respectively.^26, 27^ With increasing education level, participants tended to be younger and have higher income. In general, lifestyle behaviors, BMI, and clinical biomarkers were also socioeconomically patterned with more favorable conditions among people who had higher education level. Baseline characteristics across income levels showed similar socioeconomic patterns.

**Table 1:** Baseline characteristics of study participants*

	Type 2 diabetes cohort	Cardiovascular diseases cohort
Population	83,759	91,083
Cases	1228	3286
Incidence, %	1.5	3.6
Follow-up time, months		
Median	43	44
Interquartile	31–53	34–54
Range	13–123	13–131
Education, %		
Low	28.3	28.1
Middle	40.0	39.9
High	31.7	31.9
Household net income, %		
Low	3.1	3.2
Lower-middle	18.3	18.3
Upper-middle	30.1	29.6
High	33.2	33.3
No response or missing	15.4	15.6
Age, years	46.2±8.8	46.1±8.8
Women, %	58.7	59.0
Lifelines diet score	24.0±5.9	24.0±5.9
Total energy intake, kcal/day	2081±604	2078±605
Total alcohol intake, grams/day	4.1 (0.9, 10.5)	4.0 (0.9, 10.4)
TV watching time, hours/day	2.4±1.3	2.4±1.3
Non-occupational MVPA, minutes/week	180 (60, 360)	180 (60, 360)
Smoking status, %:		
Never	45.0	44.9
Former	35.3	34.8
Current	18.5	18.6
BMI, kg/m^2^	26.1±4.1	26.2±4.2
Fasting glucose, mmol/L	4.94±0.50	5.01±0.80
HbA_1c_, %	5.52±0.30	5.55±0.42
Triglycerides, mmol/L	1.18±0.80	1.19±0.82
HDL-cholesterol, mmol/L	1.51±0.40	1.50±0.40
Total cholesterol, mmol/L	5.17±0.97	5.16±0.98
Hypertension, %	24.6	24.2
Systolic blood pressure, mmHg	125.3±14.8	125.2±14.9
Diastolic blood pressure, mmHg	74.5±9.3	74.4±9.4
Diabetes at baseline, %		3.2
Atrial fibrillation at baseline, %		0.6

^*^Data are expressed as unadjusted mean ± standard deviation for age, Lifelines diet score (no unit, ranging from 0 to 48), total energy intake, TV watching time, BMI, fasting glucose, HbA_1c_, systolic blood pressure, diastolic blood pressure, triglycerides, HDL-cholesterol, and total cholesterol; data are expressed as median (interquartile) for total alcohol intake and non-occupational MVPA; data are expressed as observed percentage for education level, household net income level, sex (women), smoking status, hypertension, diabetes at baseline, and atrial fibrillation at baseline.

Frequency measures of incidences of type 2 diabetes and cardiovascular diseases across education and income levels are shown in Table 2. Among 83,759 participants included in the type 2 diabetes cohort, we identified 1228 cases of type 2 diabetes (incidence 1.5%) during a median follow-up of 43 months. Among 91,083 participants included in the cardiovascular diseases cohort, we identified 3286 cases of cardiovascular diseases (non-fatal cardiovascular events) during a median follow-up of 44 months. Additionally, a total of 1127 deaths were recorded during the follow-up in the cardiovascular diseases cohort. With decreasing education or income levels, incidences of type 2 diabetes and cardiovascular diseases increased. Supplementary Table S3 shows the frequency measures of incidences of type 2 diabetes and cardiovascular diseases among different combinations of education and income levels.

**Table 2:** Frequency measures of incident type 2 diabetes (a) and cardiovascular diseases (b) across education and income levels

	Cases/population	Incidence, %	Risk difference, %^*^	Proportion of cases, %^†^
(a) Type 2 diabetes				
Education				
Low	548/23,679	2.3	1.4	44.6
Middle	439/33,527	1.3	0.4	35.7
High	241/26,553	0.9	Ref.	19.6
Income				
Low	74/2606	2.8	1.8	6.0
Lower-middle	297/15,288	1.9	0.9	24.2
Upper-middle	363/25,175	1.4	0.4	29.6
High	298/27,819	1.1	Ref.	24.3
No response/missing	196/12,871	1.5	0.4	16.0
Total	1228/83,759	1.5		100
(b) Cardiovascular diseases				
Education				
Low	1170/25,636	4.6	1.7	35.6
Middle	1291/36,381	3.5	0.7	39.3
High	825/29,066	2.8	Ref.	25.1
Income				
Low	127/2928	4.3	1.2	3.9
Lower-middle	647/16,632	3.9	0.7	19.7
Upper-middle	996/26,999	3.7	0.5	30.3
High	959/30,323	3.2	Ref.	29.2
No response/missing	557/14,201	3.9	0.8	17.0
Total	3286/91,083	3.6		100

^*^Risk difference was calculated by subtracting the incidence in the reference group from the incidence in the group of interests. ^†^Proportion of cases was calculated by dividing number of cases in the group of interests by total number of cases.

Separate associations of education and income with incident type 2 diabetes and cardiovascular diseases are shown in Tables 3 and 4 and Supplementary Table S4. Low education and low income were both positively associated with higher risks of type 2 diabetes and cardiovascular diseases after adjustment for age and sex (basic model). The mutual adjustment between education and income only moderately attenuated those associations. Additional adjustment for other covariates attenuated those associations as well. In the mutually adjusted multivariate model, participants with low education had 24% (OR 1.24 [95%CI 1.04–1.48]) and 15% (OR 1.15 [95%CI 1.04–1.28]) higher odds of incident type 2 diabetes and cardiovascular diseases, respectively; participants with low income had 71% (OR 1.71 [95%CI 1.30–2.26]) and 24% (OR 1.24 [95%CI 1.02–1.52]) higher odds of incident type 2 diabetes and cardiovascular diseases, respectively, using high education and high income as reference group as appropriate. Multiplicative interactive effects between education and income were absent: OR_interaction_ 1.01 (95%CI 0.91–1.12) and OR_interaction_ 0.97 (95%CI 0.91–1.04) in multivariate models for type 2 diabetes and cardiovascular diseases, respectively. For cardiovascular diseases, similar associations were observed for the secondary composite outcome, including both non-fatal cardiovascular event and death from any cause (Supplementary Table S5).

**Table 3:** Separate associations of education and income with incident type 2 diabetes

	Basic model*		Multivariate model^†^	
	Singly adjusted^‡^	Mutually adjusted^§^	Singly adjusted^‡^	Mutually adjusted^§^
Education				
Low	2.17 (1.86–2.53)	1.85 (1.57–2.19)	1.33 (1.12–1.58)	1.24 (1.04–1.48)
Middle	1.48 (1.26–1.73)	1.37 (1.16–1.61)	1.11 (0.94–1.31)	1.07 (0.90–1.27)
High	1.00 (ref)			
Income				
Low	2.76 (2.13–3.58)	2.24 (1.71–2.92)	1.82 (1.38–2.39)	1.71 (1.30–2.26)
Lower-middle	1.76 (1.49–2.06)	1.44 (1.22–1.71)	1.30 (1.10–1.54)	1.23 (1.03–1.46)
Upper-middle	1.34 (1.15–1.56)	1.17 (1.00–1.37)	1.11 (0.95–1.29)	1.07 (0.91–1.25)
High	1.00 (ref)			

^*^Basic model: OR (95%CI) derived from multivariate logistic regression models adjusted for age and sex, *n* = 83,759 for education-singly adjusted model, and *n* = 83,381 for income-singly adjusted model and mutually adjusted model. ^†^Multivariate model: OR (95%CI) derived from multivariate logistic regression models adjusted for basic model covariates plus BMI, smoking status, TV watching time, non-occupational MVPA, total energy intake, LLDS, alcohol intake, HDL-cholesterol, triglycerides, and blood pressure, *n* = 82,908 for education-singly adjusted model, and *n* = 82,722 for income-singly adjusted model and mutually adjusted model. ^‡^Singly adjusted: models were adjusted for education and income separately. ^§^Mutually adjusted: models were adjusted for education and income simultaneously.

**Table 4:** Separate associations of education and income with incident cardiovascular diseases

	Basic model*		Multivariate model^†^	
	Singly adjusted^‡^	Mutually adjusted^§^	Singly adjusted^‡^	Mutually adjusted^§^
Education				
Low	1.42 (1.29–1.55)	1.36 (1.23–1.50)	1.17 (1.06–1.30)	1.15 (1.04–1.28)
Middle	1.31 (1.19–1.43)	1.27 (1.16–1.40)	1.18 (1.07–1.29)	1.16 (1.06–1.28)
High	1.00 (ref)			
Income				
Low	1.50 (1.23–1.81)	1.35 (1.11–1.64)	1.29 (1.06–1.58)	1.24 (1.02–1.52)
Lower-middle	1.22 (1.10–1.35)	1.10 (0.99–1.23)	1.08 (0.97–1.21)	1.04 (0.93–1.17)
Upper-middle	1.15 (1.05–1.26)	1.07 (0.97–1.18)	1.06 (0.96–1.16)	1.02 (0.93–1.13)
High	1.00 (ref)			

^*^Basic model: OR (95%CI) derived from multivariate logistic regression models adjusted for age and sex, *n* = 91,083 for education-singly adjusted model, and *n* = 90,531 for income-singly adjusted model and mutually adjusted model. ^†^Multivariate model: OR (95%CI) derived from multivariate logistic regression models adjusted for basic model covariates plus BMI, smoking status, TV watching time, non-occupational MVPA, total energy intake, LLDS, alcohol intake, HDL-cholesterol, triglycerides, total cholesterol, blood pressure, diabetes at baseline, and atrial fibrillation at baseline, *n* = 89,473 for education-singly adjusted model, and *n* = 89,251 for income-singly adjusted model and mutually adjusted model. ^‡^Singly adjusted: models were adjusted for education and income separately. ^§^Mutually adjusted: models were adjusted for education and income simultaneously.

**Figure 1:**
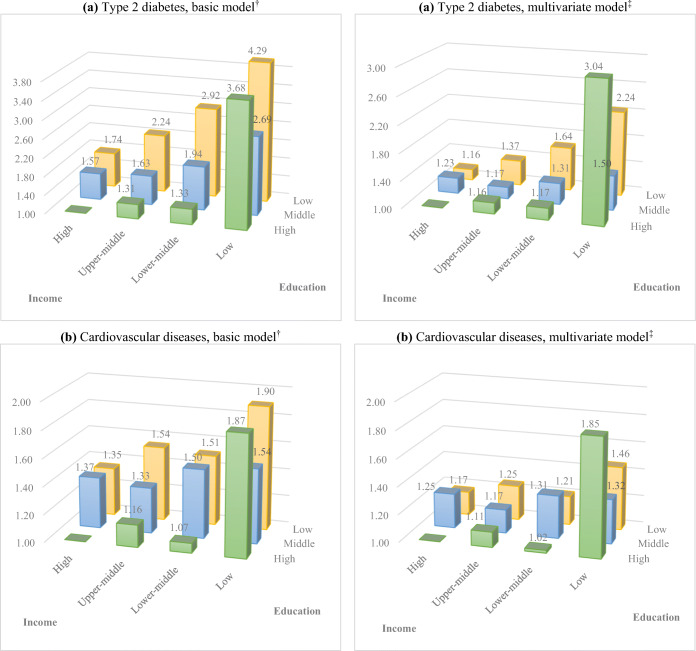
Joint associations of education and income with incident type 2 diabetes (a) and cardiovascular diseases (b). Figures are shown according to each education and income level, using high education and high income (>3000 euro/month) group as low risk reference (OR = 1.00). A dagger indicates a basic model: OR derived from multivariate logistic regression models adjusted for age and sex and *n* = 83,381 and *n* =90,531 for type 2 diabetes and cardiovascular diseases, respectively. A double dagger indicates a multivariate model: OR derived from multivariate logistic regression models adjusted for basic model covariates plus BMI, smoking status, TV watching time, non-occupational MVPA, total energy intake, LLDS, alcohol intake, HDL-cholesterol, triglycerides, blood pressure, and *n* = 82,722 and *n* = 89,251 for type 2 diabetes and cardiovascular diseases, respectively; for cardiovascular diseases, total cholesterol, diabetes at baseline, and atrial fibrillation at baseline were additionally adjusted in the multivariate model.

Joint associations of education and income with incident type 2 diabetes and cardiovascular diseases are shown in Figure 1 and Supplementary Table S6. In general, gradients of associations across education and income levels were observed after adjustment for age and sex. Further adjustment for other covariates substantially attenuated these associations. For cardiovascular diseases, gradients of associations were weakened after adjustments for these risk factors. In the multivariate model, participants who had high education and low income had the highest risks for incident type 2 diabetes (OR 3.04 [95%CI 1.52–6.05]) and cardiovascular diseases (OR 1.85 [95%CI 1.18–2.91]), followed by participants who had low education and low income, i.e., OR 2.24 [95%CI 1.54–3.25] for type 2 diabetes and OR 1.46 [95%CI 1.11–1.92] for cardiovascular diseases, using participants who had high education and high income as reference.

Percentages of attenuation in ORs across each education and income group are shown in Supplementary Table S4. When education and income were simultaneously controlled, adjustment for modifiable risk factors at baseline in total explained 33.1% and 15.2% of the associations of education with type 2 diabetes and cardiovascular diseases, respectively; for income, in total 23.5% and 7.7% of the associations were explained for type 2 diabetes and cardiovascular diseases, respectively. Adjustment for lifestyle behaviors explained more socioeconomic variations than other modifiable risk factors. Additional adjustments for clinical biomarkers and comorbid conditions at baseline (for cardiovascular diseases) showed no clear effects on explaining these socioeconomic variations.

Sensitivity analyses in general yielded similar results compared with the main analyses. Supplementary Table S7 presents the results of the analysis by including the responses of “do not know” or “prefer not to answer” for income as a single category. Large variations in the risks of incident type 2 diabetes and cardiovascular diseases were found in this income group across education levels. Furthermore, no significant multiplicative interactive effects were found for sex, age, unemployment status, comorbid conditions at baseline, and diabetes status at baseline and during the follow-up (for cardiovascular diseases), with education and income (Supplementary Table S8). For cardiovascular diseases, results were basically unchanged when adjusting for the SCORE2 risk prediction algorithms according to the European Society of Cardiology (Supplementary Table S9). For type 2 diabetes, using a composite outcome of incident type 2 diabetes and death from any cause yielded a smaller effect size for education (low education: OR 1.11 [95%CI 0.98–1.25]) but a stronger effect size for income (low income: OR 1.85 [95%CI 1.51–2.27]), compared with the main results (Supplementary Table S10).

## DISCUSSION

The primary objective of this study was to investigate the effects of education and income simultaneously on incident type 2 diabetes and cardiovascular diseases. Specifically, this study was directed at assessing the short-term inequities in incident type 2 diabetes and cardiovascular diseases. Using this large population-based cohort sample, we found that low education and low income were both independently but also differentially associated with higher risks of these two health outcomes. In addition, results from the combined associations of education and income revealed substantial health disparities in these two health outcomes across education and income levels.

In general, our results are consistent with previous similar studies on prevalent type 2 diabetes in a German cohort and incident type 2 diabetes in a US community-based cohort.^7, 28^ Our results on cardiovascular diseases are also comparable with an Italian cohort.^29^ Our analyses thus provide the very important additional evidence demonstrating the independent associations of education and income with incident type 2 diabetes and cardiovascular diseases in a European setting. With a broader perspective, we also found that our results were partly in line with studies conducted in different geographical and socioeconomic settings, despite the differences in study design and methodology that preclude direct comparisons. For example, a systematic review reported that low or middle education and low income were associated with higher risks of cardiovascular outcomes in US and European settings, while the effects of education were absent in Asian settings.^30^ On the other hand, a global study found that low education was a strong predictor for cardiovascular diseases in all 20 countries analyzed, while wealth showed no or weak associations.^31^ For type 2 diabetes, results seemed to be more consistent, as a meta-analysis found that both education and income were associated with a higher risk of developing type 2 diabetes, irrespective of different geographical settings.^32^ In brief, our study further underlines the broader notion that it is important to consider and prioritize education and income as two indispensable socioeconomic dimensions when addressing health disparities, irrespective of geographical and socioeconomic settings.^4^

The independent associations of education and income with health highlight that these two socioeconomic indicators are of equal importance and should both be considered in health research. Our findings support the hypothesis suggesting that education and income may impact on health through different causal processes, as they provide different dimensions of resources in relation to health.^1, 3, 6, 7, 28, 33–37^ More specifically, education determines one’s non-material resources such as knowledge, skills, and self-efficacy that help individuals ease their barriers to be more receptive to health messages and transfer those messages into health behaviors. Such improvements in cognitive functioning associated with higher education level were argued to be the major driver in delaying the onset of non-communicable diseases. On the other hand, income reflects one’s material resources in regard to health, such as healthy food, health services, and leisure time activities.^6, 38–40^ In line with these theoretical assumptions, we did observe that lifestyle behaviors explained a considerable proportion of the associations for both socioeconomic indicators.

Results of the combined associations of education and income indicated that their effects on health were likely to be additive. As we observed within each education or income level, substantial health disparities existed across strata of the other socioeconomic indicator. More specifically, we showed that within different education groups, a higher income was associated with a better health; similarly, within different income groups, a higher education was associated with a better health. Differences in modifiable risk factors did not fully annul these excessive risks. We further illustrate this with status inconsistency, that is, people having discrepant socioeconomic positions in two or more of these ranking indicators. For example, we observed that participants who had high income but low education were worse off regarding their health outcomes, compared with those who had a matching socioeconomic position (i.e., high income and high education). And such status inconsistency–related health disparities were prevalent across almost every education and income level in our study sample. Previous studies have shown that status inconsistency between education and occupational class carried higher health risks.^12, 13, 41^ Our findings thus provide further support of this in the dimensions of education and income. It should be noted that we observed some non-linear associations especially after adjustment for modifiable risk factors. As these non-linear associations appeared gradually with the stepwise adjustments, we were unable to clearly specify the causes of this counterintuitive finding.

In our study sample, approximately 5% of the study population had extreme status inconsistency; not surprisingly, education and income were only moderately correlated (Spearman’s correlation coefficient = 0.34, Supplementary Table S3). We therefore further emphasize the importance and necessity of considering both education and income. Especially in public health programs (such as healthy eating campaigns or diabetes screening) where the effectiveness and outreach are often compromised among people who are socioeconomically disadvantaged, additional attention and support should be given to those not only having low income, but also having low education.^42^ Additionally, further understanding of such within-group differences may lead the way toward the design of policies that do not require the adjustment of socioeconomic characteristics that are generally fixed such as education. Indeed, in our case, income supports individuals who had low education, which may contribute to their health even after their education level has been attained.

Of all modifiable risk factors examined, lifestyle behaviors and BMI contributed the most to the socioeconomic gradients, while additional adjustment for clinical biomarkers did not further explain those health disparities. The higher risk for cardiovascular diseases conferred by poor socioeconomic status also appeared to be independent of diabetes status. It is noteworthy that after accounting for all modifiable risk factors, a large proportion of the risks for type 2 diabetes and cardiovascular diseases were still left unexplained. Previous studies have found that the most socioeconomically deprived individuals had disproportionately higher risks for type 2 diabetes and cardiovascular diseases, even if they practiced the healthiest lifestyle.^2, 43^ These results indicate that even though people who have low socioeconomic status may benefit from lifestyle interventions and obesity control, their excessively higher risks of developing type 2 diabetes and cardiovascular diseases may still be preserved. Particularly, as our study was based in the context of The Netherlands, a developed country with a high coverage of government-subsidized public education system and well-structured social security system, the persistent socioeconomic patterning in health inequities observed may not be addressed only by extensive public health interventions, but also through institutional and structural changes with support in all socioeconomic dimensions simultaneously.^6^

Strengths of this study include the large sample size, which allows the investigation of joint associations of education and income with sufficient statistical power. Secondly, we also conducted sensitivity analyses supporting the robustness of our findings. On the other hand, several limitations should be noted. Because of the intrinsic limitation of the Lifelines questionnaire, we are unable to translate household net income level into individual equivalent disposable income level. Since the Lifelines cohort study was established in The Netherlands, a country with a well-developed welfare system, it may not be possible to extrapolate our results to other population groups in another setting. We are also unable to assess the possible changes in participants’ education and income level. However, education is considered to be very stable over the entire adult life. Similarly, income in The Netherlands is also relatively stable because of the organization of the Dutch labor market (e.g., wide-spread use of collective wage bargaining as well as generous unemployment insurance). We therefore do not expect dramatic changes in participants’ socioeconomic status during follow-up.^44^ Another limitation is that the resolution in time, regarding the time of diagnosis of type 2 diabetes and cardiovascular diseases, was limited in the Lifelines dataset, hence limiting the suitability of the data for survival analysis. Nevertheless, considering the low event rate, moderate effect sizes, and the relatively short follow-up time, logistic regression models may provide similar estimates for the effect sizes. We therefore used logistic regression models instead.^45, 46^ Furthermore, misclassification could occur in the ascertainment of cases of type 2 diabetes and cardiovascular diseases, as at T2 and T3 only self-reported data was available. Data on participants’ medical records and causes of death were not available in the Lifelines study. Natriuretic peptide measurements, echocardiography, and coronary imaging were not performed in the Lifelines study. For type 2 diabetes, however, we consider this lack of medical records is not expected to substantially influence our results, since at T4 most new cases were identified by objective laboratory measurements, which is a strength of our study. For cardiovascular diseases, we cross-checked new self-reported cases at T4 with electrocardiographic results. Finally, approximately 18% of the study population was excluded because of loss of follow-up. However, we do not expect this attrition to substantially influence our results. We did not observe substantial differences in the baseline characteristics between the included participants and those who had no follow-up data (Supplementary Table S11), although there seemed to be fewer participants having high education or high income among those who had no follow-up information; participants who had no follow-up data also appeared to smoke more. A simulation study found that loss to follow-up (<50%) may lead to minor underestimation on the estimates of socioeconomic inequities in cohort studies.^47^ This suggests if full information was available, our estimation would be even more pronounced, despite the clear gradients of associations that have already been revealed in our results.

## CONCLUSIONS

In conclusion, our results showed that education and income were both independently and also differentially associated with incident type 2 diabetes and cardiovascular diseases. Additionally, by analyzing the effects of education and income using a combined indicator, substantial health disparities were observed within socioeconomic groups. These findings suggest that education and income are two equally indispensable socioeconomic indicators in health, and should both be considered in health research and policymaking.

## Supplementary Information

ESM 1 (DOCX 201 kb)

## Data Availability

The manuscript is based on the data from the Lifelines cohort study. Lifelines adheres to standards for data availability. The data catalogue of the Lifelines cohort study is publicly accessible at www.lifelines.nl. All international researchers can obtain data at the Lifelines research office (research@lifelines.nl), for which a fee is required. The Lifelines research system allows access for reproducibility of the study results.

## **Abbreviations**

BMI: Body mass index
FFQ: Food frequency questionnaire
LLDS: Lifelines Diet Score
MVPA: Moderate-to-vigorous physical activity
SES: Socioeconomic status
