# Supplementary material for: Effects of Education and Income on Incident Type 2 Diabetes and Cardiovascular Diseases: a Dutch Prospective Study
Source: J Gen Intern Med. 2022 Apr 13;37(15):3907–16. doi: 10.1007/s11606-022-07548-8 (PMC9640500; doi:10.1007/s11606-022-07548-8)
Supplement: Supplementary file 1 — (DOCX 201 kb) [file 11606_2022_7548_MOESM1_ESM.docx]

**Effects of education and income on incident type 2 diabetes and cardiovascular diseases: A Dutch prospective study**

**Online Supplementary Files**

Ming-Jie Duan^1*^, Yinjie Zhu^1*^, Louise H. Dekker^1,2^, Jochen O. Mierau^2,3^, Eva Corpeleijn^4^, Stephan J.L. Bakker^1^, Gerjan Navis^1^

*^*^Joint first authors*

^1^ Department of Internal Medicine, Division of Nephrology, University Medical Centre Groningen, University of Groningen, Groningen, The Netherlands;

^2^ Aletta Jacobs School of Public Health, University of Groningen, Groningen, The Netherlands;

^3^ Faculty of Economics and Business, University of Groningen, Groningen, The Netherlands.

^4^ Department of Epidemiology, University Medical Centre Groningen, University of Groningen, Groningen, The Netherlands;

Corresponding author: Ming-Jie Duan, [m.duan@umcg.nl](mailto:m.duan@umcg.nl)

**List of Abbreviations**

**BMI** - Body mass index

**HDL** – High-density lipoprotein

**LLDS** - Lifelines Diet Score

**MVPA** - Moderate-to-vigorous physical activity

**Supplementary Figure S1** - Study design and timeline of data collection of the Lifelines cohort study


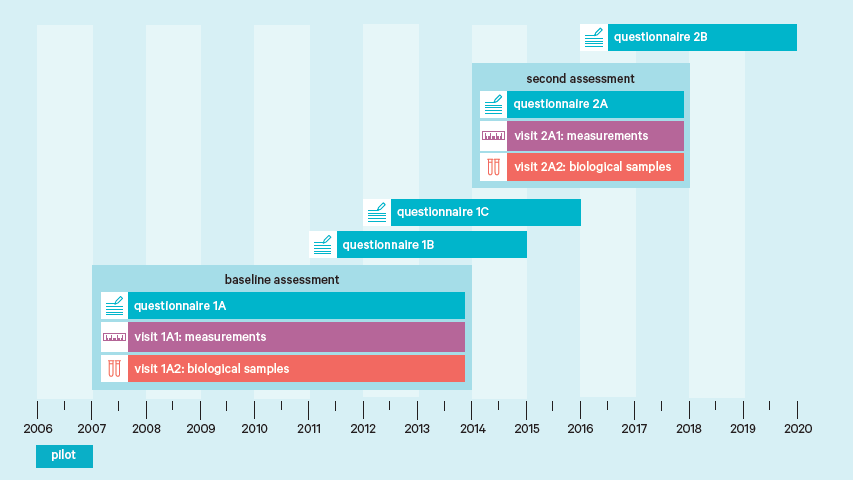


|  | Baseline assessment T1 | Follow-up T2 (questionnaire 1B) | Follow-up T3 (questionnaire 1C) | Second assessment T4 |
| --- | --- | --- | --- | --- |
| Blood samples | **Yes** | No | No | **Yes** |
| Questionnaires for status of diabetes and cardiovascular diseases | **Yes** | **Yes** | **Yes** | **Yes** |
| Electrocardiogram | **Yes** | No | No | **Yes** |

After the baseline assessment (T1, year 2007 to 2013), all participants were invited for new rounds of assessments approximately every 5 years. In between assessments, follow-up questionnaires were completed approximately once every 1.5–2.5 years. The current analysis used data from the baseline assessment T1 and the second assessment T4, as well as the two follow-ups (T2 and T3) in between. Detailed information can be found at: <https://www.lifelines.nl/researcher/data-and-biobank>

**Supplementary Figure S2** - Study flow chart for exclusions and diagnosis of new cases of type 2 diabetes and cardiovascular diseases

**(a)** Type 2 diabetes cohort

**(b)** Cardiovascular diseases cohort

**Supplementary Table S1** - Self-report questionnaires used for evaluating education (a) and income level (b) in the Lifelines cohort study

**(a)** Education level^*^

| **Question:** What is the highest degree you have obtained? | |
| --- | --- |
| **Question in Dutch:** Wat is je hoogst behaalde diploma? | |
| **Categories** | **Choices for responses** |
| High education | Higher vocational education (such as HBO, HTS, HEAO); university degree or higher (bachelor, master, doctoral degree). |
| Middle education | Secondary vocational education or work-based learning pathway (such as MBO-long, VWO, Atheneum, Gymnasium, INAS); senior general secondary education/pre-university secondary education (such as HAVO, VWO, Atheneum, Gymnasium, HBS, MMS). |
| Low education | No education (did not finish primary school); primary education (primary school, special needs primary school); lower or preparatory secondary vocational education (such as LTS, LEAO, LHNO, VMBO); junior general secondary education (such as MAVO, (M)ULO, MBO-short, VMBO-t). |
| Other, namely | If participants were not sure about their education level or did not find matching answers for their education level, they could fill out a short description of their education status or diplomas formerly obtained in the questionnaire. These string variables were further checked and re-coded manually into their corresponding education levels. |

^*^High, middle and low education levels correspond to level 0, 1, and 2 from the International Standard Classification of Education [1].

[1] UNESCO. International Standard Classification of Education - ISCED 2011. Montreal, Canada: UNESCO Institute for Statistics; 2012. Available at: <http://uis.unesco.org/sites/default/files/documents/isced-2011-en.pdf>

**(b)** Income level

| **Question:** What is the net income per month? (if you share a household, include the net income of your partner(s). | |
| --- | --- |
| **Question in Dutch:** Hoeveel bedraagt het netto inkomen per maand? (als u het huishouden met iemand deelt, dan ook de inkomsten van uw partner(s) meetellen). | |
| **Categories** | **Choices for responses** |
| Low <1000 euro/month | <750 euro/month; 750-1000 euro/month. |
| Lower-middle 1000-2000 euro/month | 1000-1500 euro/month; 1500-2000 euro/month. |
| Upper-middle 2000-3000 euro/month | 2000-2500 euro/month; 2500-3000 euro/month. |
| High >3000 euro/month | 3000-3500 euro/month; >3500 euro/month. |
| No response | I do not know/I prefer not to answer. |

**Supplementary Table S2** - Baseline characteristics of study participants according to each education and income level^*^

**(a)** Type 2 diabetes cohort by education level

|  | **Education level** | | | **Total** |
| --- | --- | --- | --- | --- |
|  | **Low** | **Middle** | **High** |  |
| Population | 23,679 | 33,527 | 26,553 | 83,759 |
| Cases | 548 | 439 | 241 | 1228 |
| Incidence, % | 2.3 | 1.3 | 0.9 | 1.5 |
| Household net income, % |  |  |  |  |
| Low | 5.1 | 3.0 | 1.4 | 3.1 |
| Lower-middle | 26.2 | 18.4 | 11.0 | 18.3 |
| Upper-middle | 31.4 | 33.9 | 24.0 | 30.1 |
| High | 15.2 | 29.0 | 54.6 | 33.2 |
| No response or missing | 22.0 | 15.8 | 8.9 | 15.4 |
| Age, years | 49.4±8.8 | 45.1±8.2 | 44.8±8.9 | 46.2±8.8 |
| Women, % | 58.9 | 61.1 | 55.6 | 58.7 |
| Lifelines diet score | 23.3±5.9 | 23.5±5.8 | 25.2±5.8 | 24.0±5.9 |
| Total energy intake, kcal/day | 2087±643 | 2095±613 | 2058±557 | 2081±604 |
| Total alcohol intake, grams/day | 3.7 (0.6-10.7) | 3.6 (0.8-10.0) | 6.0 (1.5-11.6) | 4.1 (0.9, 10.5) |
| TV watching time, hours/day | 2.9±1.4 | 2.4±1.2 | 2.0±1.1 | 2.4±1.3 |
| Non-occupational MVPA, minutes/week | 180 (60, 360) | 180 (60, 360) | 210 (90, 360) | 180 (60, 360) |
| Smoking status, %: |  |  |  |  |
| Never | 35.1 | 45.3 | 53.6 | 45.0 |
| Former | 40.0 | 34.6 | 32.1 | 35.3 |
| Current | 23.8 | 19.0 | 13.1 | 18.5 |
| BMI, kg/m^2^ | 26.8±4.3 | 26.2±4.2 | 25.3±3.7 | 26.1±4.1 |
| Fasting glucose, mmol/L | 5.02±0.52 | 4.92±0.49 | 4.88±0.48 | 4.94±0.50 |
| HbA_1c_, % | 5.58±0.30 | 5.51±0.29 | 5.48±0.29 | 5.52±0.30 |
| Triglycerides, mmol/L | 1.26±0.88 | 1.17±0.79 | 1.11±0.73 | 1.18±0.80 |
| HDL-cholesterol, mmol/L | 1.49±0.40 | 1.50±0.40 | 1.53±0.40 | 1.51±0.40 |
| Total cholesterol, mmol/L | 5.33±0.99 | 5.13±0.96 | 5.07±0.97 | 5.17±0.97 |
| Hypertension, % | 32.2 | 23.4 | 19.4 | 24.6 |
| Systolic blood pressure, mmHg | 127.9±15.4 | 125.0±14.5 | 123.3±14.5 | 125.3±14.8 |
| Diastolic blood pressure, mmHg | 75.6±9.5 | 74.4±9.2 | 73.7±9.3 | 74.5±9.3 |

^*^Data are expressed as unadjusted mean ± standard deviation for age, Lifelines diet score (no unit, ranging from 0 to 48), total energy intake, TV watching time, BMI, fasting glucose, HbA_1c_, systolic blood pressure, diastolic blood pressure, triglycerides, HDL-cholesterol, and total cholesterol; Data are expressed as median (interquartile) for total alcohol intake and non-occupational MVPA; Data are expressed as observed percentage for household net income level, sex (women), smoking status, and hypertension.

**(b)** Type 2 diabetes cohort by income level

|  | **Household monthly net income level (euro/month)** | | | | | **Total** |
| --- | --- | --- | --- | --- | --- | --- |
|  | **Low <1000** | **Lower-middle**  **1000-2000** | **Upper-middle**  **2000-3000** | **High >3000** | **No response or missing** |  |
| Population | 2606 | 15,288 | 25,175 | 27,819 | 12,871 | 83,759 |
| Cases | 74 | 297 | 363 | 298 | 196 | 1228 |
| Incidence, % | 2.8 | 1.9 | 1.4 | 1.1 | 1.5 | 1.5 |
| Education level, % |  |  |  |  |  |  |
| Low | 46.8 | 40.5 | 29.6 | 13.0 | 40.5 | 28.3 |
| Middle | 38.8 | 40.3 | 45.1 | 34.9 | 41.1 | 40.0 |
| High | 14.5 | 19.2 | 25.4 | 52.1 | 18.4 | 31.7 |
| Age, years | 46.6±9.0 | 46.8±9.5 | 46.1±8.9 | 45.5±8.3 | 47.2±8.8 | 46.2±8.8 |
| Women, % | 80.2 | 61.7 | 55.7 | 53.8 | 67.5 | 58.7 |
| Lifelines diet score | 23.4±6.3 | 23.7±6.0 | 23.8±5.8 | 24.6±5.8 | 23.5±5.9 | 24.0±5.9 |
| Total energy intake, kcal/day | 1941±604 | 2062±633 | 2120±603 | 2079±573 | 2058±635 | 2081±604 |
| Total alcohol intake, grams/day | 1.8 (0, 6.9) | 3.2 (0.5, 9.8) | 4.1 (0.9, 10.7) | 6.3 (1.7, 12.1） | 2.8 (0.4, 8.8) | 4.1 (0.9, 10.5) |
| TV watching time, hours/day | 2.9±1.7 | 2.6±1.4 | 2.4±1.3 | 2.1±1.1 | 2.5±1.3 | 2.4±1.3 |
| Non-occupational MVPA, minutes/week | 180 (60, 400) | 180 (60, 370) | 180 (60, 360) | 195 (90, 360) | 180 (60, 360) | 180 (60, 360) |
| Smoking status, %: |  |  |  |  |  |  |
| Never | 38.7 | 40.7 | 43.7 | 47.8 | 48.0 | 45.0 |
| Former | 30.6 | 34.5 | 36.5 | 36.0 | 33.4 | 35.3 |
| Current | 29.2 | 23.6 | 18.7 | 15.0 | 17.1 | 18.5 |
| BMI, kg/m^2^ | 26.4±5.1 | 26.4±4.5 | 26.2±4.0 | 25.7±3.7 | 26.3±4.3 | 26.1±4.1 |
| Fasting glucose, mmol/L | 4.92±0.52 | 4.95±0.51 | 4.95±0.50 | 4.92±0.49 | 4.93±0.50 | 4.94±0.50 |
| HbA_1c_, % | 5.54±0.31 | 5.54±0.30 | 5.52±0.30 | 5.49±0.29 | 5.54±0.30 | 5.52±0.30 |
| Triglycerides, mmol/L | 1.16±0.78 | 1.20±0.80 | 1.21±0.81 | 1.15±0.82 | 1.14±0.73 | 1.18±0.80 |
| HDL-cholesterol, mmol/L | 1.55±0.40 | 1.50±0.40 | 1.48±0.39 | 1.51±0.40 | 1.54±0.41 | 1.51±0.40 |
| Total cholesterol, mmol/L | 5.19±0.99 | 5.19±0.99 | 5.17±0.97 | 5.14±0.97 | 5.20±0.98 | 5.17±0.97 |
| Hypertension, % | 25.6 | 27.3 | 25.2 | 21.7 | 26.4 | 24.6 |
| Systolic blood pressure, mmHg | 123.5±15.7 | 125.9±15.1 | 125.8±14.8 | 124.4±14.5 | 125.5±15.0 | 125.3±14.8 |
| Diastolic blood pressure, mmHg | 73.3±9.3 | 74.6±9.4 | 74.8±9.3 | 74.4±9.3 | 74.3±9.3 | 74.5±9.3 |

^*^Data are expressed as unadjusted mean ± standard deviation for age, Lifelines diet score (no unit, ranging from 0 to 48), total energy intake, TV watching time, BMI, fasting glucose, HbA_1c_, systolic blood pressure, diastolic blood pressure, triglycerides, HDL-cholesterol, and total cholesterol; Data are expressed as median (interquartile) for total alcohol intake and non-occupational MVPA; Data are expressed as observed percentage for education level, sex (women), smoking status, and hypertension.

**(c)** Cardiovascular diseases cohort by education level

|  | **Education level** | | | **Total** |
| --- | --- | --- | --- | --- |
|  | **Low** | **Middle** | **High** |  |
| Population | 25,636 | 36,381 | 29,066 | 91,083 |
| Cases | 1170 | 1291 | 825 | 3286 |
| Incidence, % | 4.6 | 3.5 | 2.8 | 3.6 |
| Household net income, % |  |  |  |  |
| Low | 5.4 | 3.1 | 1.5 | 3.2 |
| Lower-middle | 26.1 | 18.4 | 11.1 | 18.3 |
| Upper-middle | 30.7 | 33.5 | 23.8 | 29.6 |
| High | 15.3 | 28.9 | 54.6 | 33.3 |
| No response or missing | 22.4 | 16.0 | 9.0 | 15.6 |
| Age, years | 49.3±8.8 | 45.0±8.2 | 44.6±8.9 | 46.1±8.8 |
| Women, % | 59.4 | 61.2 | 56.0 | 59.0 |
| Lifelines diet score | 23.3±5.9 | 23.5±5.8 | 25.2±5.8 | 24.0±5.9 |
| Total energy intake, kcal/day | 2084±642 | 2092±614 | 2056±560 | 2078±605 |
| Total alcohol intake, grams/day | 3.6 (0.6, 10.5) | 3.5 (0.8, 10.0) | 6.0 (1.5, 11.5) | 4.0 (0.9, 10.4) |
| TV watching time, hours/day | 2.9±1.4 | 2.4±1.2 | 2.0±1.1 | 2.4±1.3 |
| Non-occupational MVPA, minutes/week | 180 (60, 360) | 180 (60, 360) | 210 (90, 360) | 180 (60, 360) |
| Smoking status, %: |  |  |  |  |
| Never | 35.2 | 45.2 | 53.3 | 44.9 |
| Former | 39.4 | 34.0 | 31.8 | 34.8 |
| Current | 23.9 | 19.2 | 13.1 | 18.6 |
| BMI, kg/m^2^ | 27.0±4.4 | 26.3±4.3 | 25.3±3.8 | 26.2±4.2 |
| Fasting glucose, mmol/L | 5.13±0.88 | 4.99±0.78 | 4.93±0.74 | 5.01±0.80 |
| HbA_1c_, % | 5.63±0.46 | 5.54±0.41 | 5.50±0.38 | 5.55±0.42 |
| Triglycerides, mmol/L | 1.28±0.91 | 1.18±0.80 | 1.11±0.74 | 1.19±0.82 |
| HDL-cholesterol, mmol/L | 1.49±0.40 | 1.50±0.40 | 1.53±0.40 | 1.50±0.40 |
| Total cholesterol, mmol/L | 5.33±0.99 | 5.13±0.96 | 5.07±0.96 | 5.16±0.98 |
| Hypertension, % | 31.8 | 23.1 | 18.8 | 24.2 |
| Systolic blood pressure, mmHg | 128.0±15.4 | 125.0±14.5 | 123.0±14.5 | 125.2±14.9 |
| Diastolic blood pressure, mmHg | 75.6±9.5 | 74.4±9.3 | 73.5±9.3 | 74.4±9.4 |
| Diabetes at baseline, % | 4.7 | 2.9 | 2.1 | 3.2 |
| Atrial fibrillation at baseline, % | 0.8 | 0.5 | 0.5 | 0.6 |

^*^Data are expressed as unadjusted mean ± standard deviation for age, Lifelines diet score (no unit, ranging from 0 to 48), total energy intake, TV watching time, BMI, fasting glucose, HbA_1c_, systolic blood pressure, diastolic blood pressure, triglycerides, HDL-cholesterol, and total cholesterol; Data are expressed as median (interquartile) for total alcohol intake and non-occupational MVPA; Data are expressed as observed percentage for household net income level, sex (women), smoking status, hypertension, diabetes status at baseline, and atrial fibrillation status at baseline.

**(d)** Cardiovascular diseases cohort by income level

|  | **Household monthly net income level (euro/month)** | | | | | **Total** |
| --- | --- | --- | --- | --- | --- | --- |
|  | **Low <1000** | **Lower-middle**  **1000-2000** | **Upper-middle**  **2000-3000** | **High >3000** | **No response or missing** |  |
| Population | 2928 | 16,632 | 26,999 | 30,323 | 14,201 | 91,083 |
| Cases | 127 | 647 | 996 | 959 | 557 | 3286 |
| Incidence, % | 4.3 | 3.9 | 3.7 | 3.2 | 3.9 | 3.6 |
| Education level, % |  |  |  |  |  |  |
| Low | 47.1 | 40.3 | 29.2 | 12.9 | 40.5 | 28.1 |
| Middle | 38.3 | 40.4 | 45.2 | 34.7 | 41.0 | 39.9 |
| High | 14.5 | 19.3 | 25.7 | 52.3 | 18.5 | 31.9 |
| Age, years | 46.6±9.1 | 46.7±9.5 | 45.9±8.9 | 45.4±8.3 | 47.1±8.8 | 46.1±8.8 |
| Women, % | 80.6 | 62.3 | 55.9 | 54.0 | 67.6 | 59.0 |
| Lifelines diet score | 23.5±6.3 | 23.8±6.0 | 23.8±5.8 | 24.6±5.8 | 23.5±6.0 | 24.0±5.9 |
| Total energy intake, kcal/day | 1920±599 | 2060±633 | 2119±603 | 2078±576 | 2053±634 | 2078±605 |
| Total alcohol intake, grams/day | 1.8 (0, 6.9) | 3.1 (0.5, 9.8) | 4.0 (0.9, 10.5) | 6.2 (1.6, 12.0) | 2.8 (0.4, 8.7) | 4.0 (0.9, 10.4) |
| TV watching time, hours/day | 2.9±1.7 | 2.6±1.4 | 2.5±1.3 | 2.1±1.1 | 2.5±1.3 | 2.4±1.3 |
| Non-occupational MVPA, minutes/week | 190 (60, 405) | 180 (60, 370) | 180 (60, 360) | 195 (90, 360) | 180 (60, 360) | 180 (60, 360) |
| Smoking status, %: |  |  |  |  |  |  |
| Never | 39.6 | 40.5 | 43.5 | 47.8 | 47.9 | 44.9 |
| Former | 30.1 | 33.9 | 36.2 | 35.4 | 33.0 | 34.8 |
| Current | 28.2 | 23.8 | 18.8 | 15.2 | 17.2 | 18.6 |
| BMI, kg/m^2^ | 26.5±5.3 | 26.5±4.6 | 26.3±4.1 | 25.7±3.8 | 26.4±4.4 | 26.2±4.2 |
| Fasting glucose, mmol/L | 5.04±0.94 | 5.06±0.90 | 5.02±0.78 | 4.97±0.72 | 5.02±0.84 | 5.01±0.80 |
| HbA_1c_, % | 5.60±0.50 | 5.59±0.46 | 5.55±0.41 | 5.51±0.39 | 5.58±0.43 | 5.55±0.42 |
| Triglycerides, mmol/L | 1.17±0.76 | 1.22±0.83 | 1.22±0.83 | 1.16±0.83 | 1.15±0.77 | 1.19±0.82 |
| HDL-cholesterol, mmol/L | 1.55±0.41 | 1.50±0.40 | 1.48±0.39 | 1.51±0.40 | 1.54±0.41 | 1.50±0.40 |
| Total cholesterol, mmol/L | 5.19±1.00 | 5.19±0.99 | 5.16±0.97 | 5.13±0.97 | 5.20±0.98 | 5.16±0.98 |
| Hypertension, % | 26.0 | 26.8 | 24.7 | 21.3 | 26.0 | 24.2 |
| Systolic blood pressure, mmHg | 123.6±15.6 | 125.9±15.2 | 125.8±14.8 | 124.3±14.5 | 125.5±15.2 | 125.2±14.9 |
| Diastolic blood pressure, mmHg | 73.3±9.3 | 74.5±9.4 | 74.8±9.4 | 74.3±9.3 | 74.2±9.3 | 74.4±9.4 |
| Diabetes at baseline, % | 5.0 | 4.3 | 3.0 | 2.2 | 3.9 | 3.2 |
| Atrial fibrillation at baseline, % | 0.5 | 0.6 | 0.7 | 0.5 | 0.6 | 0.6 |

^*^Data are expressed as unadjusted mean ± standard deviation for age, Lifelines diet score (no unit, ranging from 0 to 48), total energy intake, TV watching time, BMI, fasting glucose, HbA_1c_, systolic blood pressure, diastolic blood pressure, triglycerides, HDL-cholesterol, and total cholesterol; Data are expressed as median (interquartile) for total alcohol intake and non-occupational MVPA; Data are expressed as observed percentage for education level, sex (women), smoking status, hypertension, diabetes status at baseline, and atrial fibrillation status at baseline.

**Supplementary Table S3** - Incidence and cases/population of type 2 diabetes (a) and cardiovascular diseases (b) (c) across education and income levels^*^

**(a)** Type 2 diabetes

| **Education** |  | **Household monthly net income** | | | | | | |
| --- | --- | --- | --- | --- | --- | --- | --- | --- |
|  |  | High | Upper-middle | Lower-middle | Low | No response | Missing |  |
| High | Incidence, % | 0.8 | 1.1 | 1.0 | 2.9 | 0.4 | Incidence = 3.1%  Cases/population = 30 / 962  Risk difference, % = 2.3 |  |
|  | Cases/population | 121 / 14,493 | 70 / 6384 | 28 / 2928 | 11 / 377 | 9 / 2259 |  |  |
|  | Risk difference | Ref. | 0.3 | 0.1 | 2.1 | -0.4 |  |  |
| Middle | Incidence, % | 1.3 | 1.3 | 1.5 | 1.9 | 1.1 |  |  |
|  | Cases/population | 122 / 9717 | 145 / 11,349 | 92 / 6166 | 19 / 1010 | 55 / 5037 |  |  |
|  | Risk difference | 0.4 | 0.4 | 0.7 | 1.0 | 0.3 |  |  |
| Low | Incidence, % | 1.5 | 2.0 | 2.9 | 3.6 | 2.2 |  |  |
|  | Cases/population | 55 / 3609 | 148 / 7442 | 177 / 6194 | 44 / 1219 | 102 / 4613 |  |  |
|  | Risk difference | 0.7 | 1.2 | 2.0 | 2.8 | 1.4 |  |  |

^*^Household monthly net income level (euro/month): high >3000, upper-middle 2000-3000, lower-middle 1000-2000, low <1000. Risk difference was calculated by subtracting the incidence in the reference group from the incidence in the group of interests.

**(b)** Cardiovascular diseases - primary outcome: first major cardiovascular event

| **Education** |  | **Household monthly net income** | | | | | |
| --- | --- | --- | --- | --- | --- | --- | --- |
|  |  | High | Upper-middle | Lower-middle | Low | No response | Missing |
| High | Incidence, % | 2.7 | 3.2 | 2.5 | 4.9 | 2.8 | Incidence = 4.9%  Cases/population = 59 / 1208  Risk difference, % = 2.2 |
|  | Cases/population | 432 / 15,871 | 222 / 6928 | 79 / 3217 | 21 / 426 | 69 / 2484 |  |
|  | Risk difference | Ref. | 0.5 | -0.3 | 2.2 | 0.1 |  |
| Middle | Incidence, % | 3.5 | 3.4 | 3.7 | 3.3 | 3.9 |  |
|  | Cases/population | 367 / 10,526 | 410 / 12,197 | 249 / 6712 | 37 / 1122 | 214 / 5522 |  |
|  | Risk difference | 0.8 | 0.6 | 1.0 | 0.6 | 1.2 |  |
| Low | Incidence, % | 4.1 | 4.6 | 4.8 | 5.0 | 4.3 |  |
|  | Cases/population | 160 / 3926 | 364 / 7874 | 319 / 6703 | 69 / 1380 | 215 / 4987 |  |
|  | Risk difference | 1.4 | 1.9 | 2.0 | 2.3 | 1.6 |  |

^*^Household monthly net income level (euro/month): high >3000, upper-middle 2000-3000, lower-middle 1000-2000, low <1000. Risk difference was calculated by subtracting the incidence in the reference group from the incidence in the group of interests.

**(c)** Cardiovascular diseases - secondary outcome: first major cardiovascular event and death from any cause

| **Education** |  | **Household monthly net income*** | | | | | |  |
| --- | --- | --- | --- | --- | --- | --- | --- | --- |
|  |  | High | Upper-middle | Lower-middle | Low | No response | Missing | Total |
| High | Incidence, % | 3.5 | 4.1 | 3.5 | 7.0 | 4.3 | Incidence = 7.1%  Cases/population = 87 / 1226  Risk difference, % = 3.6 | 3.7 |
|  | Cases/population | 549 / 15,910 | 283 / 6950 | 113 / 3235 | 30 / 429 | 107 / 2498 |  | 1087 / 29,163 |
|  | Risk difference | Ref. | 0.6 | 0.0 | 3.5 | 0.8 |  |  |
| Middle | Incidence, % | 4.4 | 4.5 | 5.0 | 4.8 | 5.1 |  | 4.7 |
|  | Cases/population | 461 / 10,562 | 551 / 12,254 | 338 / 6747 | 54 / 1129 | 286 / 5555 |  | 1709 / 36,551 |
|  | Risk difference | 0.9 | 1.0 | 1.6 | 1.3 | 1.7 |  |  |
| Low | Incidence, % | 5.2 | 6.0 | 6.9 | 7.6 | 5.9 |  | 6.3 |
|  | Cases/population | 207 / 3944 | 474 / 7927 | 471 / 6786 | 107 / 1404 | 295 / 5028 |  | 1617 / 25,870 |
|  | Risk difference | 1.8 | 2.5 | 3.5 | 4.2 | 2.4 |  |  |
| Total | Incidence, % | 4.0 | 4.8 | 5.5 | 6.4 | 5.3 | 7.1 | 4.8 |
|  | Cases/population | 1217 / 30,416 | 1308 / 27,131 | 922 / 16,768 | 191 / 2962 | 688 / 13,081 | 87 / 1226 | 4413 / 91,584 |

^*^Household monthly net income level (euro/month): high >3000, upper-middle 2000-3000, lower-middle 1000-2000, low <1000. Risk difference was calculated by subtracting the incidence in the reference group from the incidence in the group of interests.

**Supplementary Table S4** - Separate associations of education and income with incident type 2 diabetes (a) and cardiovascular diseases (b), and contribution of modifiable risk factors in explaining these associations

**(a)** Type 2 diabetes

|  | Education | | | | Income^*^ | | | | | |  |
| --- | --- | --- | --- | --- | --- | --- | --- | --- | --- | --- | --- |
|  | Low | | Middle | | Low | | Lower-middle | | Upper-middle | | Reference |
| Models**^†^** | OR (95%CI) | %At**^‡^** | OR (95%CI) | %At**^‡^** | OR (95%CI) | %At**^‡^** | OR (95%CI) | %At**^‡^** | OR (95%CI) | %At**^‡^** | models |
| Basic model | 1.85 (1.57-2.19) |  | 1.37 (1.16-1.61) |  | 2.24 (1.71-2.92) |  | 1.44 (1.22-1.71) |  | 1.17 (1.00-1.37) |  |  |
| Model 2 | 1.46 (1.23-1.75) | 20.9 | 1.20 (1.01-1.42) | 12.2 | 1.91 (1.46-2.51) | 14.6 | 1.33 (1.11-1.58) | 8.1 | 1.13 (0.97-1.33) | 3.2 | Basic model |
| Model 3 | 1.43 (1.21-1.69) | 22.7 | 1.15 (0.97-1.36) | 15.7 | 1.95 (1.48-2.56) | 13.0 | 1.31 (1.10-1.56) | 9.4 | 1.11 (0.94-1.30) | 5.5 | Basic model |
| Model 4 | 1.50 (1.27-1.78) | 19.0 | 1.20 (1.02-1.42) | 11.8 | 2.01 (1.53-2.63) | 10.4 | 1.33 (1.12-1.58) | 7.9 | 1.10 (0.94-1.29) | 6.0 | Basic model |
| Model 5 | 1.25 (1.05-1.49) | 32.5 | 1.07 (0.90-1.27) | 21.6 | 1.75 (1.33-2.32) | 21.6 | 1.24 (1.04-1.48) | 14.0 | 1.09 (0.93-1.28) | 6.9 | Basic model |
| Multivariate model | 1.24 (1.04-1.48) | 33.1 | 1.07 (0.90-1.27) | 21.5 | 1.71 (1.30-2.26) | 23.5 | 1.23 (1.03-1.46) | 15.0 | 1.07 (0.91-1.25) | 8.9 | Basic model |
|  |  | 0.9 |  | -0.1 |  | 2.4 |  | 1.2 |  | 2.1 | Model 5 |

^*^Household monthly net income level (euro/month): high >3000, upper-middle 2000-3000, lower-middle 1000-2000, low <1000. ^†^All models were estimated using multivariate logistic regression models, using high education and high income as reference (OR = 1.00). Education and income were mutually adjusted in all models. Basic model was adjusted for age and sex, *n* = 83,381. Model 2 was adjusted for basic model covariates plus lifestyle behaviors (smoking status, TV watching time, non-occupational MVPA, total energy intake, LLDS, and alcohol intake), *n* = 82,753. Model 3 was adjusted for basic model covariates plus BMI, *n* = 83,381. Model 4 was adjusted for basic model covariates plus clinical biomarkers (HDL-cholesterol, triglycerides, and blood pressure), *n* = 83,342. Model 5 was adjusted for basic model covariates plus lifestyle behaviors and BMI, *n* = 82,753. Multivariate model was adjusted for model 5 covariates plus clinical biomarkers, *n* = 82,722. ^†^Attenuation, %: contributions of each domain of modifiable risk factors in explaining the associations of income and education with incident type 2 diabetes were determined by calculating the percentage of attenuation in the ORs after additional adjustment for another set of modifiable risk factors, in comparison to the previous reference model, namely 100×(OR_ref_-OR_new_)/OR_ref_.

**(b)** Cardiovascular diseases

|  | Education | | | | | Income^*^ | | | | | | |  | |
| --- | --- | --- | --- | --- | --- | --- | --- | --- | --- | --- | --- | --- | --- | --- |
|  | Low | | Middle | | Low | | | Lower-middle | | Upper-middle | | Reference | |  |
| Models**^†^** | OR (95%CI) | %At**^‡^** | OR (95%CI) | %At**^‡^** | OR (95%CI) | | %At**^‡^** | OR (95%CI) | %At**^‡^** | OR (95%CI) | %At**^‡^** | models | |  |
| Basic model | 1.36 (1.23-1.50) |  | 1.27 (1.16-1.40) |  | 1.35 (1.11-1.64) | |  | 1.10 (0.99-1.23) |  | 1.07 (0.97-1.18) |  |  | |  |
| Model 2 | 1.22 (1.10-1.36) | 9.8 | 1.21 (1.10-1.33) | 5.3 | 1.26 (1.03-1.54) | | 6.9 | 1.07 (0.95-1.19) | 3.2 | 1.05 (0.95-1.15) | 2.1 | Basic model | |  |
| Model 3 | 1.26 (1.14-1.39) | 7.2 | 1.21 (1.11-1.33) | 4.7 | 1.31 (1.08-1.60) | | 2.6 | 1.08 (0.97-1.21) | 1.8 | 1.06 (0.96-1.16) | 1.1 | Basic model | |  |
| Model 4 | 1.25 (1.13-1.38) | 8.1 | 1.22 (1.11-1.33) | 4.7 | 1.33 (1.09-1.63) | | 1.1 | 1.08 (0.97-1.21) | 1.7 | 1.05 (0.95-1.16) | 1.7 | Basic model | |  |
| Model 5 | 1.17 (1.05-1.30) | 14.1 | 1.17 (1.06-1.28) | 8.4 | 1.24 (1.01-1.52) | | 8.2 | 1.05 (0.94-1.18) | 4.2 | 1.04 (0.94-1.15) | 2.8 | Basic model | |  |
| Model 6 | 1.15 (1.03-1.27) | 1.8 | 1.16 (1.05-1.27) | 1.0 | 1.25 (1.02-1.53) | | -0.9 | 1.05 (0.94-1.17) | 0.5 | 1.03 (0.93-1.14) | 0.8 | Model 5 | |  |
| Multivariate model | 1.15 (1.04-1.28) | 15.2 | 1.16 (1.06-1.28) | 8.7 | 1.24 (1.02-1.52) | | 7.7 | 1.04 (0.93-1.17) | 5.3 | 1.02 (0.93-1.13) | 4.2 | Basic model | |  |
|  |  | -0.5 |  | -0.6 |  |  | 0.4 |  | 0.5 |  | 0.7 | Model 6 | |  |

^*^Household monthly net income level (euro/month): high >3000, upper-middle 2000-3000, lower-middle 1000-2000, low <1000. ^†^All models were estimated using multivariate logistic regression models, using high education and high income as reference (OR = 1.00). Education and income were mutually adjusted in all models. Basic model was adjusted for age and sex, *n* = 90,531. Model 2 was adjusted for basic model covariates plus lifestyle behaviors (smoking status, TV watching time, non-occupational MVPA, total energy intake, LLDS, and alcohol intake), *n* = 89,538. Model 3 was adjusted for basic model covariates plus BMI, *n* = 90,531. Model 4 was adjusted for basic model covariates plus clinical biomarkers (HDL-cholesterol, triglycerides, total cholesterol, and blood pressure), *n* = 89,875. Model 5 was adjusted for basic model covariates plus lifestyle behaviors and BMI, *n* = 89,538. Model 6 was adjusted for model 5 covariates plus clinical biomarkers, *n* = 89,251. Multivariate model was adjusted for model 6 covariates plus diabetes and atrial fibrillation at baseline, *n* = 89,251. ^†^Attenuation, %: contributions of each domain of modifiable risk factors in explaining the associations of income and education with cardiovascular diseases were determined by calculating the percentage of attenuation in the ORs after additional adjustment for another set of modifiable risk factors, in comparison to the previous reference model, namely 100×(OR_ref_-OR_new_)/OR_ref_.

**Supplementary Table S5** - Separate (a) and combined (b) associations of education and income with the secondary composite outcome of cardiovascular diseases: the earliest non-fatal cardiovascular event and death from any cause

**(a)** Separate associations

|  | **Basic model^*^** | **Multivariate model^†^** |
| --- | --- | --- |
| Education |  |  |
| Low | 1.35 (1.23-1.47) | 1.13 (1.03-1.24) |
| Middle | 1.26 (1.17-1.37) | 1.15 (1.05-1.25) |
| High | 1.00 (ref) | |
| Income |  |  |
| Low | 1.53 (1.29-1.81) | 1.38 (1.15-1.64) |
| Lower-middle | 1.20 (1.09-1.31) | 1.12 (1.01-1.23) |
| Upper-middle | 1.09 (1.01-1.19) | 1.05 (0.96-1.14) |
| High | 1.00 (ref) | |

^*^Basic model: OR (95% CI) derived from multivariate logistic regression models adjusted for age and sex, *n* = 91,016. ^†^Multivariate model: OR (95% CI) derived from multivariate logistic regression models adjusted for basic model covariates plus BMI, smoking status, TV watching time, non-occupational MVPA, total energy intake, LLDS, alcohol intake, HDL-cholesterol, triglycerides, total cholesterol, blood pressure, diabetes, and atrial fibrillation, *n* = 89,701.

**(b)** Combined associations

| **Education** | High | Middle | Low | High | Middle | Low |
| --- | --- | --- | --- | --- | --- | --- |
| **Income** | High >3000 euro/month | | | Upper-middle 2000-3000 euro/month | | |
| Basic model^*^ | 1.00 (ref) | 1.33 (1.18-1.51) | 1.35 (1.15-1.58) | 1.15 (1.00-1.33) | 1.38 (1.23-1.55) | 1.52 (1.35-1.71) |
| Multivariate model^†^ |  | 1.20 (1.06-1.36) | 1.15 (0.97-1.35) | 1.10 (0.94-1.27) | 1.20 (1.07-1.36) | 1.22 (1.07-1.38) |
| **Education** | High | Middle | Low | High | Middle | Low |
| **Income** | Lower-middle 1000-2000 euro/month | | | Low <1000 euro/month | | |
| Basic model^*^ | 1.18 (0.96-1.45) | 1.57 (1.38-1.80) | 1.64 (1.45-1.85) | 2.08 (1.42-3.05) | 1.70 (1.28-2.27) | 2.13 (1.71-2.66) |
| Multivariate model^†^ | 1.11 (0.89-1.37) | 1.33 (1.16-1.53) | 1.28 (1.12-1.46) | 2.04 (1.38-3.01) | 1.41 (1.05-1.90) | 1.57 (1.24-1.98) |

^*^Basic model: OR (95% CI) derived from multivariate logistic regression models adjusted for age and sex, *n* = 91,016. ^†^Multivariate model: OR (95% CI) derived from multivariate logistic regression models adjusted for basic model covariates plus BMI, smoking status, TV watching time, non-occupational MVPA, total energy intake, LLDS, alcohol intake, HDL-cholesterol, triglycerides, total cholesterol, blood pressure, diabetes, and atrial fibrillation, *n* = 89,701.

**Supplementary Table S6** - Combined associations of education and income with incident type 2 diabetes (a) and cardiovascular diseases (b)^*^

**(a)** Type 2 diabetes

| **Education** | High | Middle | Low | High | Middle | Low |
| --- | --- | --- | --- | --- | --- | --- |
| **Income** | High >3000 euro/month | | | Upper-middle 2000-3000 euro/month | | |
| Model 1 | 1.00 (ref) | 1.57 (1.23-2.00) | 1.74 (1.28-2.36) | 1.31 (0.97-1.76) | 1.63 (1.29-2.07) | 2.24 (1.76-2.83) |
| Model 2 |  | 1.38 (1.07-1.77) | 1.39 (1.02-1.90) | 1.26 (0.94-1.70) | 1.39 (1.09-1.78) | 1.72 (1.34-2.20) |
| Model 3 |  | 1.25 (0.97-1.61) | 1.19 (0.86-1.63) | 1.21 (0.90-1.64) | 1.20 (0.94-1.54) | 1.42 (1.11-1.82) |
| Model 4 |  | 1.23 (0.95-1.58) | 1.16 (0.85-1.59) | 1.16 (0.86-1.57) | 1.17 (0.91-1.50) | 1.37 (1.07-1.76) |
| **Education** | High | Middle | Low | High | Middle | Low |
| **Income** | Lower-middle 1000-2000 euro/month | | | Low <1000 euro/month | | |
| Model 1 | 1.33 (0.88-2.00) | 1.94 (1.49-2.53) | 2.92 (2.31-3.68) | 3.68 (1.96-6.91) | 2.69 (1.65-4.38) | 4.29 (3.02-6.08) |
| Model 2 | 1.25 (0.83-1.88) | 1.58 (1.20-2.07) | 2.12 (1.66-2.71) | 3.18 (1.69-5.95) | 1.99 (1.20-3.30) | 2.92 (2.02-4.20) |
| Model 3 | 1.21 (0.80-1.84) | 1.31 (1.00-1.73) | 1.70 (1.33-2.17) | 3.07 (1.59-5.92) | 1.59 (0.95-2.64) | 2.32 (1.59-3.36) |
| Model 4 | 1.17 (0.76-1.78) | 1.31 (0.99-1.72) | 1.64 (1.28-2.10) | 3.04 (1.52-6.05) | 1.50 (0.89-2.51) | 2.24 (1.54-3.25) |

^*^For all models, multivariate logistic regression was applied to estimate the ORs (95%CI). Model 1 was adjusted for age and sex, *n* = 83,381; Model 2 was adjusted for model 1 covariates plus lifestyle behaviors (smoking status, TV watching time, non-occupational moderate-to-vigorous physical activity, total energy intake, Lifelines diet score, and alcohol intake), *n* = 82,753; Model 3 was adjusted for Model 2 covariates plus BMI, *n* = 82,753; Model 4 was adjusted for Model 3 covariates plus clinical biomarkers (HDL-cholesterol, triglycerides, and blood pressure), *n* = 82,722.

**(b)** Cardiovascular diseases

| **Education** | High | Middle | Low | High | Middle | Low |
| --- | --- | --- | --- | --- | --- | --- |
| **Income** | High >3000 euro/month | | | Upper-middle 2000-3000 euro/month | | |
| Model 1 | 1.00 (ref) | 1.37 (1.20-1.57) | 1.35 (1.13-1.62) | 1.16 (0.98-1.37) | 1.33 (1.16-1.53) | 1.54 (1.34-1.76) |
| Model 2 |  | 1.29 (1.13-1.48) | 1.23 (1.03-1.48) | 1.13 (0.96-1.33) | 1.24 (1.08-1.42) | 1.36 (1.18-1.57) |
| Model 3 |  | 1.26 (1.09-1.44) | 1.18 (0.98-1.42) | 1.12 (0.95-1.32) | 1.19 (1.04-1.37) | 1.29 (1.12-1.49) |
| Model 4 |  | 1.24 (1.08-1.42) | 1.16 (0.97-1.39) | 1.11 (0.94-1.31) | 1.17 (1.02-1.35) | 1.25 (1.08-1.45) |
| Model 5 |  | 1.25 (1.08-1.43) | 1.17 (0.97-1.40) | 1.11 (0.94-1.31) | 1.17 (1.02-1.34) | 1.25 (1.08-1.45) |
| **Education** | High | Middle | Low | High | Middle | Low |
| **Income** | Lower-middle 1000-2000 euro/month | | | Low <1000 euro/month | | |
| Model 1 | 1.07 (0.84-1.37) | 1.50 (1.29-1.76) | 1.51 (1.31-1.74) | 1.87 (1.20-2.93) | 1.54 (1.09-2.18) | 1.90 (1.45-2.47) |
| Model 2 | 1.05 (0.82-1.34) | 1.38 (1.18-1.62) | 1.31 (1.13-1.53) | 1.80 (1.15-2.82) | 1.37 (0.96-1.94) | 1.58 (1.20-2.07) |
| Model 3 | 1.06 (0.83-1.35) | 1.32 (1.13-1.55) | 1.24 (1.06-1.44) | 1.81 (1.15-2.85) | 1.30 (0.91-1.86) | 1.48 (1.13-1.95) |
| Model 4 | 1.04 (0.81-1.33) | 1.30 (1.11-1.53) | 1.21 (1.04-1.41) | 1.83 (1.16-2.88) | 1.30 (0.91-1.85) | 1.46 (1.11-1.93) |
| Model 5 | 1.02 (0.80-1.32) | 1.31 (1.11-1.54) | 1.21 (1.04-1.41) | 1.85 (1.18-2.91) | 1.32 (0.93-1.87) | 1.46 (1.11-1.92) |

^*^For all models, multivariate logistic regression was applied to estimate the ORs (95%CI). Model 1 was adjusted for age and sex, *n* = 90,531; Model 2 was adjusted for model 1 covariates plus lifestyle behaviors (smoking status, TV watching time, non-occupational moderate-to-vigorous physical activity, total energy intake, Lifelines diet score, and alcohol intake), *n* = 89,538; Model 3 was adjusted for Model 2 covariates plus BMI, *n* = 89,538; Model 4 was adjusted for Model 3 covariates plus clinical biomarkers (HDL-cholesterol, triglycerides, total cholesterol, and blood pressure), *n* = 89,251; Model 5 was adjusted for model 4 covariates plus status of atrial fibrillation and diabetes at baseline, *n* = 89,251.

**Supplementary Table S7** - Associations of education and income with incident type 2 diabetes (a, b) and cardiovascular diseases (c, d), without imputation of income

**(a)** Type 2 diabetes - separate associations

|  | **Basic model^*^** | **Multivariate model^†^** | |
| --- | --- | --- | --- |
| Education |  |  | |
| Low | 1.90 (1.61-2.24) | 1.26 (1.06-1.51) | |
| Middle | 1.38 (1.18-1.63) | 1.09 (0.92-1.29) | |
| High | 1.00 (ref) | | |
| Income |  |  | |
| Low | 2.35 (1.80-3.08) | 1.78 (1.34-2.36) | |
| Lower-middle | 1.44 (1.21-1.70) | 1.22 (1.02-1.46) | |
| Upper-middle | 1.15 (0.98-1.35) | 1.05 (0.89-1.23) | |
| High | 1.00 (ref) | | |
| No response | 1.08 (0.89-1.31) | 0.98 (0.80-1.19) |  |

**(b)** Type 2 diabetes - combined associations

| **Education** | High | Middle | Low | High | Middle | Low |
| --- | --- | --- | --- | --- | --- | --- |
| **Income** | High >3000 euro/month | | | Upper-middle 2000-3000 euro/month | | |
| Basic model^*^ | 1.00 (ref) | 1.55 (1.20-1.99) | 1.58 (1.14-2.18) | 1.32 (0.98-1.78) | 1.59 (1.25-2.03) | 2.07 (1.62-2.64) |
| Multivariate model^†^ |  | 1.21 (0.93-1.56) | 1.06 (0.76-1.48) | 1.17 (0.86-1.58) | 1.14 (0.88-1.46) | 1.28 (0.99-1.66) |
| **Education** | High | Middle | Low | High | Middle | Low |
| **Income** | Lower-middle 1000-2000 euro/month | | | Low <1000 euro/month | | |
| Basic model^*^ | 1.34 (0.89-2.03) | 1.92 (1.46-2.52) | 2.84 (2.24-3.61) | 3.77 (2.01-7.08) | 2.70 (1.65-4.40) | 4.49 (3.15-6.40) |
| Multivariate model^†^ | 1.18 (0.77-1.81) | 1.28 (0.96-1.70) | 1.60 (1.24-2.06) | 3.08 (1.54-6.15) | 1.46 (0.87-2.46) | 2.33 (1.60-3.39) |
| **Education** | High | Middle | Low |  | | |
| **Income** | No response | | |  |  |  |
| Basic model^*^ | 0.50 (0.26-0.99) | 1.39 (1.01-1.91) | 2.41 (1.84-3.16) |  |  |  |
| Multivariate model^†^ | 0.49 (0.24-0.96) | 1.05 (0.76-1.47) | 1.38 (1.04-1.85) |  |  |  |

^*^Basic model: OR (95% CI) derived from multivariate logistic regression models adjusted for age and sex, *n* = 82,797. ^†^Multivariate model: OR (95% CI) derived from multivariate logistic regression models adjusted for basic model covariates plus BMI, smoking status, TV watching time, non-occupational MVPA, total energy intake, LLDS, alcohol intake, HDL-cholesterol, triglycerides, and blood pressure, *n* = 81,981.

**(c)** Cardiovascular diseases - separate associations

|  | **Basic model^*^** | **Multivariate model^†^** | |
| --- | --- | --- | --- |
| Education |  |  | |
| Low | 1.34 (1.21-1.48) | 1.13 (1.02-1.26) | |
| Middle | 1.26 (1.15-1.39) | 1.15 (1.05-1.27) | |
| High | 1.00 (ref) | | |
| Income |  |  | |
| Low | 1.42 (1.17-1.73) | 1.32 (1.08-1.61) | |
| Lower-middle | 1.10 (0.99-1.23) | 1.05 (0.94-1.17) | |
| Upper-middle | 1.07 (0.98-1.18) | 1.03 (0.93-1.13) | |
| High | 1.00 (ref) | | |
| No response | 1.14 (1.02-1.28) | 1.10 (0.98-1.24) |  |

**(d)** Cardiovascular diseases - combined associations

| **Education** | High | Middle | Low | High | Middle | Low |
| --- | --- | --- | --- | --- | --- | --- |
| **Income** | High >3000 euro/month | | | Upper-middle 2000-3000 euro/month | | |
| Basic model^*^ | 1.00 (ref) | 1.34 (1.17-1.55) | 1.35 (1.12-1.62) | 1.18 (1.00-1.39) | 1.30 (1.13-1.49) | 1.53 (1.33-1.77) |
| Multivariate model^†^ |  | 1.21 (1.05-1.40) | 1.16 (0.96-1.41) | 1.12 (0.95-1.33) | 1.13 (0.98-1.31) | 1.25 (1.07-1.45) |
| **Education** | High | Middle | Low | High | Middle | Low |
| **Income** | Lower-middle 1000-2000 euro/month | | | Low <1000 euro/month | | |
| Basic model^*^ | 1.07 (0.84-1.37) | 1.49 (1.27-1.75) | 1.48 (1.28-1.72) | 1.97 (1.26-3.10) | 1.52 (1.08-2.15) | 2.01 (1.54-2.62) |
| Multivariate model^†^ | 1.03 (0.80-1.32) | 1.30 (1.10-1.53) | 1.19 (1.02-1.39) | 1.96 (1.24-3.09) | 1.30 (0.91-1.84) | 1.54 (1.17-2.03) |
| **Education** | High | Middle | Low |  | | |
| **Income** | No response | | |  |  |  |
| Basic model^*^ | 1.10 (0.85-1.43) | 1.57 (1.32-1.85) | 1.51 (1.27-1.79) |  |  |  |
| Multivariate model^†^ | 1.04 (0.79-1.36) | 1.40 (1.17-1.66) | 1.23 (1.03-1.46) |  |  |  |

^*^Basic model: OR (95% CI) derived from multivariate logistic regression models adjusted for age and sex, *n* = 89,875. ^†^Multivariate model: OR (95% CI) derived from multivariate logistic regression models adjusted for basic model covariates plus BMI, smoking status, TV watching time, non-occupational MVPA, total energy intake, LLDS, alcohol intake, HDL-cholesterol, triglycerides, total cholesterol, blood pressure, diabetes, and atrial fibrillation, *n* = 88,324.

**Supplementary Table S8** - Effects of age (a), sex (b), unemployment status (c), diabetes status (d), cardiovascular diseases status (e), and cancer status (f) on the associations of education and income with incident type 2 diabetes and cardiovascular diseases

**(a)** Age^*^

| **Type 2 diabetes (*n* = 82,722)** | | | | |
| --- | --- | --- | --- | --- |
| **Interaction terms** | 30-39 years | 40-49 years | 50-59 years | 60-65 years |
| Education | 0.85 (0.63-1.16) | 0.93 (0.76-1.16) | 1.15 (0.92-1.42) | 1.00 (ref) |
| Income | 0.81 (0.61-1.08) | 0.90 (0.74-1.10) | 0.99 (0.81-1.23) | 1.00 (ref) |

| **Cardiovascular diseases (*n* = 89,251)** | | | | |
| --- | --- | --- | --- | --- |
| **Interaction terms** | 30-39 years | 40-49 years | 50-59 years | 60-65 years |
| Education | 0.94 (0.79-1.12) | 0.93 (0.82-1.06) | 0.97 (0.85-1.11) | 1.00 (ref) |
| Income | 0.99 (0.85-1.15) | 1.02 (0.90-1.15) | 0.99 (0.87-1.12) | 1.00 (ref) |

^*^All models were estimated using logistic regression adjusted for age, sex, BMI, smoking status, TV watching time, non-occupational MVPA, total energy intake, LLDS, alcohol intake, HDL-cholesterol, triglycerides, and blood pressure. Total cholesterol, diabetes, and atrial fibrillation were additionally adjusted for cardiovascular diseases. Education and income were mutually adjusted in all models. Multiplicative interactive effects between age and education or income were estimated by entering an interaction term in the model, results are shown as ORs (95%CI).

**(b)** Sex^*^

|  | **Type 2 diabetes (*n* = 82,722)** | | **Cardiovascular diseases (*n* =89,251 )** | |
| --- | --- | --- | --- | --- |
| **Interaction terms** | Women | Men | Women | Men |
| Education | 1.00 (ref) | 1.05 (0.89-1.24) | 1.00 (ref) | 0.91 (0.82-1.00) |
| Income | 1.00 (ref) | 1.03 (0.88-1.20) | 1.00 (ref) | 1.01 (0.92-1.11) |

^*^All models were estimated using logistic regression adjusted for age, sex, BMI, smoking status, TV watching time, non-occupational MVPA, total energy intake, LLDS, alcohol intake, HDL-cholesterol, triglycerides, and blood pressure. Total cholesterol, diabetes, and atrial fibrillation were additionally adjusted for cardiovascular diseases. Education and income were mutually adjusted in all models. Multiplicative interactive effects between sex and education or income were estimated by entering an interaction term in the model, results are shown as ORs (95%CI).

**(c)** Unemployment status^*^

| **No interaction term** | **Type 2 diabetes (*n* = 82,722)** | **Cardiovascular diseases (*n* = 89,251)** |
| --- | --- | --- |
| Education |  |  |
| Low | 1.24 (1.04-1.48) | 1.15 (1.03-1.28) |
| Middle | 1.07 (0.90-1.27) | 1.16 (1.06-1.28) |
| High | 1.00 (ref) | |
| Income |  |  |
| Low | 1.70 (1.29-2.25) | 1.26 (1.03-1.55) |
| Lower-middle | 1.22 (1.02-1.46) | 1.05 (0.93-1.17) |
| Upper-middle | 1.07 (0.91-1.25) | 1.03 (0.93-1.13) |
| High | 1.00 (ref) | |
| Unemployment Status |  |  |
| No | 1.05 (0.78-1.42) | 0.89 (0.72-1.10) |
| Yes | 1.00 (ref) | |

| **Interaction term** | **Type 2 diabetes (*n* = 82,722)** | | **Cardiovascular diseases (*n* = 89,251)** | |
| --- | --- | --- | --- | --- |
|  | Unemployed | No | Unemployed | No |
| Education | 1.00 (ref) | 0.80 (0.54-1.19) | 1.00 (ref) | 0.86 (0.65-1.14) |
| Income | 1.00 (ref) | 1.13 (0.82-1.54) | 1.00 (ref) | 0.94 (0.75-1.18) |

^*^All models were estimated using logistic regression adjusted for age, sex, BMI, smoking status, TV watching time, non-occupational MVPA, total energy intake, LLDS, alcohol intake, HDL-cholesterol, triglycerides, blood pressure, and unemployment status. Total cholesterol, diabetes, and atrial fibrillation were additionally adjusted for cardiovascular diseases. Education and income were mutually adjusted in all models. Multiplicative interactive effects between unemployment status and education or income were estimated by entering an interaction term in the model, results are shown as ORs (95%CI).

**(d)** Cardiovascular diseases - diabetes status (*n* = 81,873)^*^

| **No interaction term** |  |
| --- | --- |
| Education |  |
| Low | 1.13 (1.01-1.26) |
| Middle | 1.16 (1.05-1.28) |
| High | 1.00 (ref) |
| Income |  |
| Low | 1.27 (1.03-1.57) |
| Lower-middle | 1.06 (0.94-1.19) |
| Upper-middle | 1.00 (0.91-1.11) |
| High | 1.00 (ref) |
| Diabetes status |  |
| Developed diabetes during follow-up | 1.57 (1.24-1.98) |
| Diabetes at baseline | 1.40 (1.20-1.64) |
| No diabetes | 1.00 (ref) |

| **Interaction term** | No diabetes | Developed diabetes during follow-up | Diabetes at baseline |
| --- | --- | --- | --- |
| Education | 1.15 (0.93-1.43) | 1.06 (0.70-1.61) | 1.00 (ref) |
| Income | 1.03 (0.85-1.25) | 1.02 (0.72-1.44) | 1.00 (ref) |

^*^All models were estimated using logistic regression adjusted for age, sex, BMI, smoking status, TV watching time, non-occupational MVPA, total energy intake, LLDS, alcohol intake, HDL-cholesterol, triglycerides, blood pressure, total cholesterol, diabetes status at baseline and during follow-up, and atrial fibrillation at baseline. Education and income were mutually adjusted in all models. Multiplicative interactive effects between diabetes status and education or income were estimated by entering an interaction term in the model, results are shown as ORs (95%CI).

**(e)** Type 2 diabetes - cardiovascular diseases history (*n* = 82,722)^*^

| **No interaction term** |  |
| --- | --- |
| Education |  |
| Low | 1.23 (1.03-1.48) |
| Middle | 1.07 (0.91-1.27) |
| High | 1.00 (ref) |
| Income |  |
| Low | 1.69 (1.28-2.23) |
| Lower-middle | 1.22 (1.02-1.45) |
| Upper-middle | 1.06 (0.91-1.25) |
| High | 1.00 (ref) |
| Cardiovascular events history at baseline | 1.47 (1.20-1.81) |
| No cardiovascular events history at baseline | 1.00 (ref) |

| **Interaction term** | Cardiovascular events history at baseline | No cardiovascular events history at baseline |
| --- | --- | --- |
| Education | 0.93 (0.70-1.22) | 1.00 (ref) |
| Income | 0.96 (0.76-1.23) | 1.00 (ref) |

^*^All models were estimated using logistic regression adjusted for age, sex, BMI, smoking status, TV watching time, non-occupational MVPA, total energy intake, LLDS, alcohol intake, HDL-cholesterol, triglycerides, blood pressure, and cardiovascular events history at baseline. Education and income were mutually adjusted in all models. Multiplicative interactive effects between diabetes status and education or income were estimated by entering an interaction term in the model, results are shown as ORs (95%CI).

**(f-1)** Type 2 diabetes - cancer status at baseline (*n* = 82,662)^*^

| **No interaction term** |  |
| --- | --- |
| Education |  |
| Low | 1.23 (1.03-1.47) |
| Middle | 1.07 (0.90-1.27) |
| High | 1.00 (ref) |
| Income |  |
| Low | 1.72 (1.30-2.27) |
| Lower-middle | 1.22 (1.02-1.46) |
| Upper-middle | 1.07 (0.91-1.25) |
| High | 1.00 (ref) |
| No cancer at baseline | 1.45 (1.06-1.98) |
| Having cancer at baseline | 1.00 (ref) |

| **Interaction term** | No cancer at baseline | Having cancer at baseline |
| --- | --- | --- |
| Education | 0.83 (0.53-1.29) | 1.00 (ref) |
| Income | 0.90 (0.61-1.33) | 1.00 (ref) |

^*^All models were estimated using logistic regression adjusted for age, sex, BMI, smoking status, TV watching time, non-occupational MVPA, total energy intake, LLDS, alcohol intake, HDL-cholesterol, triglycerides, blood pressure, and cancer status at baseline. Education and income were mutually adjusted in all models. Multiplicative interactive effects between diabetes status and education or income were estimated by entering an interaction term in the model, results are shown as ORs (95%CI).

**(f-2)** Cardiovascular diseases - cancer status at baseline (*n* = 89,173)^*^

| **No interaction term** |  |
| --- | --- |
| Education |  |
| Low | 1.15 (1.03-1.28) |
| Middle | 1.16 (1.06-1.28) |
| High | 1.00 (ref) |
| Income |  |
| Low | 1.25 (1.02-1.53) |
| Lower-middle | 1.05 (0.93-1.17) |
| Upper-middle | 1.02 (0.93-1.13) |
| High | 1.00 (ref) |
| No cancer at baseline | 0.92 (0.78-1.08) |
| Having cancer at baseline | 1.00 (ref) |

| **Interaction term** | No cancer at baseline | Having cancer at baseline |
| --- | --- | --- |
| Education | 0.88 (0.70-1.09) | 1.00 (ref) |
| Income | 1.00 (0.82-1.23) | 1.00 (ref) |

^*^All models were estimated using logistic regression adjusted for age, sex, BMI, smoking status, TV watching time, non-occupational MVPA, total energy intake, LLDS, alcohol intake, HDL-cholesterol, triglycerides, blood pressure, total cholesterol, cancer status at baseline, and atrial fibrillation at baseline. Education and income were mutually adjusted in all models. Multiplicative interactive effects between diabetes status and education or income were estimated by entering an interaction term in the model, results are shown as ORs (95%CI).

**Supplementary Table S9 -** Effects of education and income on incident cardiovascular diseases, with adjustment for SCORE2 risk prediction algorithms according to European Society of Cardiology^*^

|  | **SCORE2 risk prediction algorithms** |
| --- | --- |
| Education |  |
| Low | 1.21 (1.09-1.34) |
| Middle | 1.21 (1.10-1.33) |
| High | 1.00 (ref) |
| Income |  |
| Low | 1.32 (1.08-1.61) |
| Lower-middle | 1.08 (0.97-1.21) |
| Upper-middle | 1.06 (0.96-1.16) |
| High | 1.00 (ref) |

^*^Models were estimated using logistic regression adjusted for sex and SCORE 2 risk prediction algorithms, *n* = 89,843. Education and income were mutually adjusted in models. Results are shown as ORs (95%CI).

Reference: SCORE2 working group and ESC Cardiovascular risk collaboration, SCORE2 risk prediction algorithms: new models to estimate 10-year risk of cardiovascular disease in Europe, European Heart Journal, Volume 42, Issue 25, 1 July 2021, Pages 2439–2454, <https://doi.org/10.1093/eurheartj/ehab309>

**Supplementary Table S10** - Associations of education and income with a composite outcome of type 2 diabetes: incident type 2 diabetes and death from any cause (*n* = 83,268)^*^

|  | **Incident type 2 diabetes and death from any cause** |
| --- | --- |
| Education |  |
| Low | 1.11 (0.98-1.25) |
| Middle | 1.06 (0.94-1.19) |
| High | 1.00 (ref.) |
| Income |  |
| Low | 1.85 (1.51-2.27) |
| Lower-middle | 1.30 (1.14-1.49) |
| Upper-middle | 1.08 (0.96-1.22) |
| High | 1.00 (ref.) |

^*^Models were estimated using logistic regression adjusted for age, sex, BMI, smoking status, TV watching time, non-occupational MVPA, total energy intake, LLDS, alcohol intake, HDL-cholesterol, triglycerides, total cholesterol, and blood pressure. Results are shown as ORs (95%CI).

**Supplementary Table S11 -** Baseline characteristics of participants who had no follow-up data^*^

|  | **Type 2 diabetes cohort** | **Cardiovascular diseases cohort** |
| --- | --- | --- |
| Population | 26,841 | 21,300 |
| Age, years | 44.3±8.6 | 43.2±8.2 |
| Sex, women % | 57.7 | 57.8 |
| Education, % |  |  |
| Low | 33.5 | 34.5 |
| Middle | 39.1 | 39.1 |
| High | 26.8 | 25.0 |
| Household net income, % |  |  |
| Low | 4.5 | 4.6 |
| Lower-middle | 19.2 | 19.6 |
| Upper-middle | 26.3 | 25.9 |
| High | 29.4 | 27.4 |
| No response or missing | 19.8 | 21.4 |
| Invalid income questionnaire | 0.9 | 1.1 |
| BMI, kg/m^2^ | 26.4±4.4 | 26.6±4.6 |
| Fasting glucose, mmol/L | 4.93±0.51 | 5.03±0.85 |
| HbA_1c_, % | 5.50±0.30 | 5.54±0.44 |
| Systolic blood pressure, mmHg | 124.4±14.9 | 124.9±15.0 |
| Diastolic blood pressure, mmHg | 74.2±9.5 | 74.3±9.4 |
| Triglycerides, mmol/L | 1.20±0.87 | 1.23±0.93 |
| HDL-cholesterol, mmol/L | 1.48±0.40 | 1.46±0.39 |
| Total cholesterol, mmol/L | 5.14±0.99 | 5.12±0.99 |
| Total energy intake, kcal/day | 2082±646 | 2088±658 |
| Total alcohol intake, grams/day | 3.6 (0.8, 10.3) | 3.4 (0.7, 10.1) |
| TV watching, hours/day | 2.5±1.4 | 2.5±1.4 |
| Non-occupational MVPA, minutes/week | 180 (60, 360) | 150 (40, 330) |
| Smoking status, %: |  |  |
| Never | 41.4 | 36.2 |
| Former | 30.2 | 25.6 |
| Current | 26.3 | 24.9 |
| Diabetes at baseline, % | / | 3.3 |
| Atrial fibrillation at baseline, % | / | 0.6 |

^*^Data are expressed as unadjusted mean ± standard deviation for age, BMI, fasting glucose, HbA_1c_, systolic blood pressure, diastolic blood pressure, triglycerides, HDL-cholesterol, total cholesterol, total energy intake, and TV watching time; Data are expressed as median (interquartile) for total alcohol intake and non-occupational moderate-to-vigorous physical activity (MVPA); Data are expressed as observed percentage for education level, household monthly net income level, sex, smoking status, diabetes at baseline, and atrial fibrillation at baseline. Lifelines diet score (LLDS) was not calculated and compared, since this score is derived on a relative scale.
